# Search for new phenomena in high-mass final states with a photon and a jet from $pp$ collisions at $\sqrt{s}$ = 13 TeV with the ATLAS detector

**DOI:** 10.1140/epjc/s10052-018-5553-2

**Published:** 2018-02-03

**Authors:** M. Aaboud, G. Aad, B. Abbott, O. Abdinov, B. Abeloos, S. H. Abidi, O. S. AbouZeid, N. L. Abraham, H. Abramowicz, H. Abreu, R. Abreu, Y. Abulaiti, B. S. Acharya, S. Adachi, L. Adamczyk, J. Adelman, M. Adersberger, T. Adye, A. A. Affolder, Y. Afik, T. Agatonovic-Jovin, C. Agheorghiesei, J. A. Aguilar-Saavedra, S. P. Ahlen, F. Ahmadov, G. Aielli, S. Akatsuka, H. Akerstedt, T. P. A. Åkesson, E. Akilli, A. V. Akimov, G. L. Alberghi, J. Albert, P. Albicocco, M. J. Alconada Verzini, S. C. Alderweireldt, M. Aleksa, I. N. Aleksandrov, C. Alexa, G. Alexander, T. Alexopoulos, M. Alhroob, B. Ali, M. Aliev, G. Alimonti, J. Alison, S. P. Alkire, B. M. M. Allbrooke, B. W. Allen, P. P. Allport, A. Aloisio, A. Alonso, F. Alonso, C. Alpigiani, A. A. Alshehri, M. I. Alstaty, B. Alvarez Gonzalez, D. Álvarez Piqueras, M. G. Alviggi, B. T. Amadio, Y. Amaral Coutinho, C. Amelung, D. Amidei, S. P. Amor Dos Santos, S. Amoroso, G. Amundsen, C. Anastopoulos, L. S. Ancu, N. Andari, T. Andeen, C. F. Anders, J. K. Anders, K. J. Anderson, A. Andreazza, V. Andrei, S. Angelidakis, I. Angelozzi, A. Angerami, A. V. Anisenkov, N. Anjos, A. Annovi, C. Antel, M. Antonelli, A. Antonov, D. J. Antrim, F. Anulli, M. Aoki, L. Aperio Bella, G. Arabidze, Y. Arai, J. P. Araque, V. Araujo Ferraz, A. T. H. Arce, R. E. Ardell, F. A. Arduh, J-F. Arguin, S. Argyropoulos, M. Arik, A. J. Armbruster, L. J. Armitage, O. Arnaez, H. Arnold, M. Arratia, O. Arslan, A. Artamonov, G. Artoni, S. Artz, S. Asai, N. Asbah, A. Ashkenazi, L. Asquith, K. Assamagan, R. Astalos, M. Atkinson, N. B. Atlay, K. Augsten, G. Avolio, B. Axen, M. K. Ayoub, G. Azuelos, A. E. Baas, M. J. Baca, H. Bachacou, K. Bachas, M. Backes, P. Bagnaia, M. Bahmani, H. Bahrasemani, J. T. Baines, M. Bajic, O. K. Baker, P. J. Bakker, E. M. Baldin, P. Balek, F. Balli, W. K. Balunas, E. Banas, A. Bandyopadhyay, Sw. Banerjee, A. A. E. Bannoura, L. Barak, E. L. Barberio, D. Barberis, M. Barbero, T. Barillari, M-S Barisits, J. T. Barkeloo, T. Barklow, N. Barlow, S. L. Barnes, B. M. Barnett, R. M. Barnett, Z. Barnovska-Blenessy, A. Baroncelli, G. Barone, A. J. Barr, L. Barranco Navarro, F. Barreiro, J. Barreiro Guimarães da Costa, R. Bartoldus, A. E. Barton, P. Bartos, A. Basalaev, A. Bassalat, R. L. Bates, S. J. Batista, J. R. Batley, M. Battaglia, M. Bauce, F. Bauer, H. S. Bawa, J. B. Beacham, M. D. Beattie, T. Beau, P. H. Beauchemin, P. Bechtle, H. P. Beck, H. C. Beck, K. Becker, M. Becker, C. Becot, A. J. Beddall, A. Beddall, V. A. Bednyakov, M. Bedognetti, C. P. Bee, T. A. Beermann, M. Begalli, M. Begel, J. K. Behr, A. S. Bell, G. Bella, L. Bellagamba, A. Bellerive, M. Bellomo, K. Belotskiy, O. Beltramello, N. L. Belyaev, O. Benary, D. Benchekroun, M. Bender, N. Benekos, Y. Benhammou, E. Benhar Noccioli, J. Benitez, D. P. Benjamin, M. Benoit, J. R. Bensinger, S. Bentvelsen, L. Beresford, M. Beretta, D. Berge, E. Bergeaas Kuutmann, N. Berger, J. Beringer, S. Berlendis, N. R. Bernard, G. Bernardi, C. Bernius, F. U. Bernlochner, T. Berry, P. Berta, C. Bertella, G. Bertoli, I. A. Bertram, C. Bertsche, D. Bertsche, G. J. Besjes, O. Bessidskaia Bylund, M. Bessner, N. Besson, A. Bethani, S. Bethke, A. J. Bevan, J. Beyer, R. M. Bianchi, O. Biebel, D. Biedermann, R. Bielski, K. Bierwagen, N. V. Biesuz, M. Biglietti, T. R. V. Billoud, H. Bilokon, M. Bindi, A. Bingul, C. Bini, S. Biondi, T. Bisanz, C. Bittrich, D. M. Bjergaard, J. E. Black, K. M. Black, R. E. Blair, T. Blazek, I. Bloch, C. Blocker, A. Blue, W. Blum, U. Blumenschein, S. Blunier, G. J. Bobbink, V. S. Bobrovnikov, S. S. Bocchetta, A. Bocci, C. Bock, M. Boehler, D. Boerner, D. Bogavac, A. G. Bogdanchikov, C. Bohm, V. Boisvert, P. Bokan, T. Bold, A. S. Boldyrev, A. E. Bolz, M. Bomben, M. Bona, M. Boonekamp, A. Borisov, G. Borissov, J. Bortfeldt, D. Bortoletto, V. Bortolotto, D. Boscherini, M. Bosman, J. D. Bossio Sola, J. Boudreau, J. Bouffard, E. V. Bouhova-Thacker, D. Boumediene, C. Bourdarios, S. K. Boutle, A. Boveia, J. Boyd, I. R. Boyko, A. J. Bozson, J. Bracinik, A. Brandt, G. Brandt, O. Brandt, F. Braren, U. Bratzler, B. Brau, J. E. Brau, W. D. Breaden Madden, K. Brendlinger, A. J. Brennan, L. Brenner, R. Brenner, S. Bressler, D. L. Briglin, T. M. Bristow, D. Britton, D. Britzger, F. M. Brochu, I. Brock, R. Brock, G. Brooijmans, T. Brooks, W. K. Brooks, J. Brosamer, E. Brost, J. H Broughton, P. A. Bruckman de Renstrom, D. Bruncko, A. Bruni, G. Bruni, L. S. Bruni, S. Bruno, BH Brunt, M. Bruschi, N. Bruscino, P. Bryant, L. Bryngemark, T. Buanes, Q. Buat, P. Buchholz, A. G. Buckley, I. A. Budagov, F. Buehrer, M. K. Bugge, O. Bulekov, D. Bullock, T. J. Burch, S. Burdin, C. D. Burgard, A. M. Burger, B. Burghgrave, K. Burka, S. Burke, I. Burmeister, J. T. P. Burr, E. Busato, D. Büscher, V. Büscher, P. Bussey, J. M. Butler, C. M. Buttar, J. M. Butterworth, P. Butti, W. Buttinger, A. Buzatu, A. R. Buzykaev, S. Cabrera Urbán, D. Caforio, H. Cai, V. M. Cairo, O. Cakir, N. Calace, P. Calafiura, A. Calandri, G. Calderini, P. Calfayan, G. Callea, L. P. Caloba, S. Calvente Lopez, D. Calvet, S. Calvet, T. P. Calvet, R. Camacho Toro, S. Camarda, P. Camarri, D. Cameron, R. Caminal Armadans, C. Camincher, S. Campana, M. Campanelli, A. Camplani, A. Campoverde, V. Canale, M. Cano Bret, J. Cantero, T. Cao, M. D. M. Capeans Garrido, I. Caprini, M. Caprini, M. Capua, R. M. Carbone, R. Cardarelli, F. Cardillo, I. Carli, T. Carli, G. Carlino, B. T. Carlson, L. Carminati, R. M. D. Carney, S. Caron, E. Carquin, S. Carrá, G. D. Carrillo-Montoya, D. Casadei, M. P. Casado, M. Casolino, D. W. Casper, R. Castelijn, V. Castillo Gimenez, N. F. Castro, A. Catinaccio, J. R. Catmore, A. Cattai, J. Caudron, V. Cavaliere, E. Cavallaro, D. Cavalli, M. Cavalli-Sforza, V. Cavasinni, E. Celebi, F. Ceradini, L. Cerda Alberich, A. S. Cerqueira, A. Cerri, L. Cerrito, F. Cerutti, A. Cervelli, S. A. Cetin, A. Chafaq, D. Chakraborty, S. K. Chan, W. S. Chan, Y. L. Chan, P. Chang, J. D. Chapman, D. G. Charlton, C. C. Chau, C. A. Chavez Barajas, S. Che, S. Cheatham, A. Chegwidden, S. Chekanov, S. V. Chekulaev, G. A. Chelkov, M. A. Chelstowska, C. Chen, C. Chen, H. Chen, J. Chen, S. Chen, S. Chen, X. Chen, Y. Chen, H. C. Cheng, H. J. Cheng, A. Cheplakov, E. Cheremushkina, R. Cherkaoui El Moursli, E. Cheu, K. Cheung, L. Chevalier, V. Chiarella, G. Chiarelli, G. Chiodini, A. S. Chisholm, A. Chitan, Y. H. Chiu, M. V. Chizhov, K. Choi, A. R. Chomont, S. Chouridou, Y. S. Chow, V. Christodoulou, M. C. Chu, J. Chudoba, A. J. Chuinard, J. J. Chwastowski, L. Chytka, A. K. Ciftci, D. Cinca, V. Cindro, I. A. Cioara, A. Ciocio, F. Cirotto, Z. H. Citron, M. Citterio, M. Ciubancan, A. Clark, B. L. Clark, M. R. Clark, P. J. Clark, R. N. Clarke, C. Clement, Y. Coadou, M. Cobal, A. Coccaro, J. Cochran, L. Colasurdo, B. Cole, A. P. Colijn, J. Collot, T. Colombo, P. Conde Muiño, E. Coniavitis, S. H. Connell, I. A. Connelly, S. Constantinescu, G. Conti, F. Conventi, M. Cooke, A. M. Cooper-Sarkar, F. Cormier, K. J. R. Cormier, M. Corradi, F. Corriveau, A. Cortes-Gonzalez, G. Costa, M. J. Costa, D. Costanzo, G. Cottin, G. Cowan, B. E. Cox, K. Cranmer, S. J. Crawley, R. A. Creager, G. Cree, S. Crépé-Renaudin, F. Crescioli, W. A. Cribbs, M. Cristinziani, V. Croft, G. Crosetti, A. Cueto, T. Cuhadar Donszelmann, A. R. Cukierman, J. Cummings, M. Curatolo, J. Cúth, S. Czekierda, P. Czodrowski, G. D’amen, S. D’Auria, L. D’eramo, M. D’Onofrio, M. J. Da Cunha Sargedas De Sousa, C. Da Via, W. Dabrowski, T. Dado, T. Dai, O. Dale, F. Dallaire, C. Dallapiccola, M. Dam, J. R. Dandoy, M. F. Daneri, N. P. Dang, A. C. Daniells, N. S. Dann, M. Danninger, M. Dano Hoffmann, V. Dao, G. Darbo, S. Darmora, J. Dassoulas, A. Dattagupta, T. Daubney, W. Davey, C. David, T. Davidek, D. R. Davis, P. Davison, E. Dawe, I. Dawson, K. De, R. de Asmundis, A. De Benedetti, S. De Castro, S. De Cecco, N. De Groot, P. de Jong, H. De la Torre, F. De Lorenzi, A. De Maria, D. De Pedis, A. De Salvo, U. De Sanctis, A. De Santo, K. De Vasconcelos Corga, J. B. De Vivie De Regie, R. Debbe, C. Debenedetti, D. V. Dedovich, N. Dehghanian, I. Deigaard, M. Del Gaudio, J. Del Peso, D. Delgove, F. Deliot, C. M. Delitzsch, A. Dell’Acqua, L. Dell’Asta, M. Dell’Orso, M. Della Pietra, D. della Volpe, M. Delmastro, C. Delporte, P. A. Delsart, D. A. DeMarco, S. Demers, M. Demichev, A. Demilly, S. P. Denisov, D. Denysiuk, D. Derendarz, J. E. Derkaoui, F. Derue, P. Dervan, K. Desch, C. Deterre, K. Dette, M. R. Devesa, P. O. Deviveiros, A. Dewhurst, S. Dhaliwal, F. A. Di Bello, A. Di Ciaccio, L. Di Ciaccio, W. K. Di Clemente, C. Di Donato, A. Di Girolamo, B. Di Girolamo, B. Di Micco, R. Di Nardo, K. F. Di Petrillo, A. Di Simone, R. Di Sipio, D. Di Valentino, C. Diaconu, M. Diamond, F. A. Dias, M. A. Diaz, E. B. Diehl, J. Dietrich, S. Díez Cornell, A. Dimitrievska, J. Dingfelder, P. Dita, S. Dita, F. Dittus, F. Djama, T. Djobava, J. I. Djuvsland, M. A. B. do Vale, D. Dobos, M. Dobre, D. Dodsworth, C. Doglioni, J. Dolejsi, Z. Dolezal, M. Donadelli, S. Donati, P. Dondero, J. Donini, J. Dopke, A. Doria, M. T. Dova, A. T. Doyle, E. Drechsler, M. Dris, Y. Du, J. Duarte-Campderros, A. Dubreuil, E. Duchovni, G. Duckeck, A. Ducourthial, O. A. Ducu, D. Duda, A. Dudarev, A. Chr. Dudder, E. M. Duffield, L. Duflot, M. Dührssen, C. Dulsen, M. Dumancic, A. E. Dumitriu, A. K. Duncan, M. Dunford, A. Duperrin, H. Duran Yildiz, M. Düren, A. Durglishvili, D. Duschinger, B. Dutta, D. Duvnjak, M. Dyndal, B. S. Dziedzic, C. Eckardt, K. M. Ecker, R. C. Edgar, T. Eifert, G. Eigen, K. Einsweiler, T. Ekelof, M. El Kacimi, R. El Kosseifi, V. Ellajosyula, M. Ellert, S. Elles, F. Ellinghaus, A. A. Elliot, N. Ellis, J. Elmsheuser, M. Elsing, D. Emeliyanov, Y. Enari, O. C. Endner, J. S. Ennis, M. B. Epland, J. Erdmann, A. Ereditato, M. Ernst, S. Errede, M. Escalier, C. Escobar, B. Esposito, O. Estrada Pastor, A. I. Etienvre, E. Etzion, H. Evans, A. Ezhilov, M. Ezzi, F. Fabbri, L. Fabbri, V. Fabiani, G. Facini, R. M. Fakhrutdinov, S. Falciano, R. J. Falla, J. Faltova, Y. Fang, M. Fanti, A. Farbin, A. Farilla, C. Farina, E. M. Farina, T. Farooque, S. Farrell, S. M. Farrington, P. Farthouat, F. Fassi, P. Fassnacht, D. Fassouliotis, M. Faucci Giannelli, A. Favareto, W. J. Fawcett, L. Fayard, O. L. Fedin, W. Fedorko, S. Feigl, L. Feligioni, C. Feng, E. J. Feng, M. J. Fenton, A. B. Fenyuk, L. Feremenga, P. Fernandez Martinez, S. Fernandez Perez, J. Ferrando, A. Ferrari, P. Ferrari, R. Ferrari, D. E. Ferreira de Lima, A. Ferrer, D. Ferrere, C. Ferretti, F. Fiedler, A. Filipčič, M. Filipuzzi, F. Filthaut, M. Fincke-Keeler, K. D. Finelli, M. C. N. Fiolhais, L. Fiorini, A. Fischer, C. Fischer, J. Fischer, W. C. Fisher, N. Flaschel, I. Fleck, P. Fleischmann, R. R. M. Fletcher, T. Flick, B. M. Flierl, L. R. Flores Castillo, M. J. Flowerdew, G. T. Forcolin, A. Formica, F. A. Förster, A. Forti, A. G. Foster, D. Fournier, H. Fox, S. Fracchia, P. Francavilla, M. Franchini, S. Franchino, D. Francis, L. Franconi, M. Franklin, M. Frate, M. Fraternali, D. Freeborn, S. M. Fressard-Batraneanu, B. Freund, D. Froidevaux, J. A. Frost, C. Fukunaga, T. Fusayasu, J. Fuster, O. Gabizon, A. Gabrielli, A. Gabrielli, G. P. Gach, S. Gadatsch, S. Gadomski, G. Gagliardi, L. G. Gagnon, C. Galea, B. Galhardo, E. J. Gallas, B. J. Gallop, P. Gallus, G. Galster, K. K. Gan, S. Ganguly, Y. Gao, Y. S. Gao, F. M. Garay Walls, C. García, J. E. García Navarro, J. A. García Pascual, M. Garcia-Sciveres, R. W. Gardner, N. Garelli, V. Garonne, A. Gascon Bravo, K. Gasnikova, C. Gatti, A. Gaudiello, G. Gaudio, I. L. Gavrilenko, C. Gay, G. Gaycken, E. N. Gazis, C. N. P. Gee, J. Geisen, M. Geisen, M. P. Geisler, K. Gellerstedt, C. Gemme, M. H. Genest, C. Geng, S. Gentile, C. Gentsos, S. George, D. Gerbaudo, G. Geßner, S. Ghasemi, M. Ghneimat, B. Giacobbe, S. Giagu, N. Giangiacomi, P. Giannetti, S. M. Gibson, M. Gignac, M. Gilchriese, D. Gillberg, G. Gilles, D. M. Gingrich, M. P. Giordani, F. M. Giorgi, P. F. Giraud, P. Giromini, G. Giugliarelli, D. Giugni, F. Giuli, C. Giuliani, M. Giulini, B. K. Gjelsten, S. Gkaitatzis, I. Gkialas, E. L. Gkougkousis, P. Gkountoumis, L. K. Gladilin, C. Glasman, J. Glatzer, P. C. F. Glaysher, A. Glazov, M. Goblirsch-Kolb, J. Godlewski, S. Goldfarb, T. Golling, D. Golubkov, A. Gomes, R. Gonçalo, R. Goncalves Gama, J. Goncalves Pinto Firmino Da Costa, G. Gonella, L. Gonella, A. Gongadze, S. González de la Hoz, S. Gonzalez-Sevilla, L. Goossens, P. A. Gorbounov, H. A. Gordon, I. Gorelov, B. Gorini, E. Gorini, A. Gorišek, A. T. Goshaw, C. Gössling, M. I. Gostkin, C. A. Gottardo, C. R. Goudet, D. Goujdami, A. G. Goussiou, N. Govender, E. Gozani, I. Grabowska-Bold, P. O. J. Gradin, J. Gramling, E. Gramstad, S. Grancagnolo, V. Gratchev, P. M. Gravila, C. Gray, H. M. Gray, Z. D. Greenwood, C. Grefe, K. Gregersen, I. M. Gregor, P. Grenier, K. Grevtsov, J. Griffiths, A. A. Grillo, K. Grimm, S. Grinstein, Ph. Gris, J.-F. Grivaz, S. Groh, E. Gross, J. Grosse-Knetter, G. C. Grossi, Z. J. Grout, A. Grummer, L. Guan, W. Guan, J. Guenther, F. Guescini, D. Guest, O. Gueta, B. Gui, E. Guido, T. Guillemin, S. Guindon, U. Gul, C. Gumpert, J. Guo, W. Guo, Y. Guo, R. Gupta, S. Gupta, S. Gurbuz, G. Gustavino, B. J. Gutelman, P. Gutierrez, N. G. Gutierrez Ortiz, C. Gutschow, C. Guyot, M. P. Guzik, C. Gwenlan, C. B. Gwilliam, A. Haas, C. Haber, H. K. Hadavand, N. Haddad, A. Hadef, S. Hageböck, M. Hagihara, H. Hakobyan, M. Haleem, J. Haley, G. Halladjian, G. D. Hallewell, K. Hamacher, P. Hamal, K. Hamano, A. Hamilton, G. N. Hamity, P. G. Hamnett, L. Han, S. Han, K. Hanagaki, K. Hanawa, M. Hance, B. Haney, P. Hanke, J. B. Hansen, J. D. Hansen, M. C. Hansen, P. H. Hansen, K. Hara, A. S. Hard, T. Harenberg, F. Hariri, S. Harkusha, P. F. Harrison, N. M. Hartmann, Y. Hasegawa, A. Hasib, S. Hassani, S. Haug, R. Hauser, L. Hauswald, L. B. Havener, M. Havranek, C. M. Hawkes, R. J. Hawkings, D. Hayakawa, D. Hayden, C. P. Hays, J. M. Hays, H. S. Hayward, S. J. Haywood, S. J. Head, T. Heck, V. Hedberg, L. Heelan, S. Heer, K. K. Heidegger, S. Heim, T. Heim, B. Heinemann, J. J. Heinrich, L. Heinrich, C. Heinz, J. Hejbal, L. Helary, A. Held, S. Hellman, C. Helsens, R. C. W. Henderson, Y. Heng, S. Henkelmann, A. M. Henriques Correia, S. Henrot-Versille, G. H. Herbert, H. Herde, V. Herget, Y. Hernández Jiménez, H. Herr, G. Herten, R. Hertenberger, L. Hervas, T. C. Herwig, G. G. Hesketh, N. P. Hessey, J. W. Hetherly, S. Higashino, E. Higón-Rodriguez, K. Hildebrand, E. Hill, J. C. Hill, K. H. Hiller, S. J. Hillier, M. Hils, I. Hinchliffe, M. Hirose, D. Hirschbuehl, B. Hiti, O. Hladik, X. Hoad, J. Hobbs, N. Hod, M. C. Hodgkinson, P. Hodgson, A. Hoecker, M. R. Hoeferkamp, F. Hoenig, D. Hohn, T. R. Holmes, M. Homann, S. Honda, T. Honda, T. M. Hong, B. H. Hooberman, W. H. Hopkins, Y. Horii, A. J. Horton, J-Y. Hostachy, A. Hostiuc, S. Hou, A. Hoummada, J. Howarth, J. Hoya, M. Hrabovsky, J. Hrdinka, I. Hristova, J. Hrivnac, T. Hryn’ova, A. Hrynevich, P. J. Hsu, S.-C. Hsu, Q. Hu, S. Hu, Y. Huang, Z. Hubacek, F. Hubaut, F. Huegging, T. B. Huffman, E. W. Hughes, G. Hughes, M. Huhtinen, R. F. H. Hunter, P. Huo, N. Huseynov, J. Huston, J. Huth, R. Hyneman, G. Iacobucci, G. Iakovidis, I. Ibragimov, L. Iconomidou-Fayard, Z. Idrissi, P. Iengo, O. Igonkina, T. Iizawa, Y. Ikegami, M. Ikeno, Y. Ilchenko, D. Iliadis, N. Ilic, F. Iltzsche, G. Introzzi, P. Ioannou, M. Iodice, K. Iordanidou, V. Ippolito, M. F. Isacson, N. Ishijima, M. Ishino, M. Ishitsuka, C. Issever, S. Istin, F. Ito, J. M. Iturbe Ponce, R. Iuppa, H. Iwasaki, J. M. Izen, V. Izzo, S. Jabbar, P. Jackson, R. M. Jacobs, V. Jain, K. B. Jakobi, K. Jakobs, S. Jakobsen, T. Jakoubek, D. O. Jamin, D. K. Jana, R. Jansky, J. Janssen, M. Janus, P. A. Janus, G. Jarlskog, N. Javadov, T. Javůrek, M. Javurkova, F. Jeanneau, L. Jeanty, J. Jejelava, A. Jelinskas, P. Jenni, C. Jeske, S. Jézéquel, H. Ji, J. Jia, H. Jiang, Y. Jiang, Z. Jiang, S. Jiggins, J. Jimenez Pena, S. Jin, A. Jinaru, O. Jinnouchi, H. Jivan, P. Johansson, K. A. Johns, C. A. Johnson, W. J. Johnson, K. Jon-And, R. W. L. Jones, S. D. Jones, S. Jones, T. J. Jones, J. Jongmanns, P. M. Jorge, J. Jovicevic, X. Ju, A. Juste Rozas, M. K. Köhler, A. Kaczmarska, M. Kado, H. Kagan, M. Kagan, S. J. Kahn, T. Kaji, E. Kajomovitz, C. W. Kalderon, A. Kaluza, S. Kama, A. Kamenshchikov, N. Kanaya, L. Kanjir, V. A. Kantserov, J. Kanzaki, B. Kaplan, L. S. Kaplan, D. Kar, K. Karakostas, N. Karastathis, M. J. Kareem, E. Karentzos, S. N. Karpov, Z. M. Karpova, K. Karthik, V. Kartvelishvili, A. N. Karyukhin, K. Kasahara, L. Kashif, R. D. Kass, A. Kastanas, Y. Kataoka, C. Kato, A. Katre, J. Katzy, K. Kawade, K. Kawagoe, T. Kawamoto, G. Kawamura, E. F. Kay, V. F. Kazanin, R. Keeler, R. Kehoe, J. S. Keller, E. Kellermann, J. J. Kempster, J Kendrick, H. Keoshkerian, O. Kepka, B. P. Kerševan, S. Kersten, R. A. Keyes, M. Khader, F. Khalil-zada, A. Khanov, A. G. Kharlamov, T. Kharlamova, A. Khodinov, T. J. Khoo, V. Khovanskiy, E. Khramov, J. Khubua, S. Kido, C. R. Kilby, H. Y. Kim, S. H. Kim, Y. K. Kim, N. Kimura, O. M. Kind, B. T. King, D. Kirchmeier, J. Kirk, A. E. Kiryunin, T. Kishimoto, D. Kisielewska, V. Kitali, O. Kivernyk, E. Kladiva, T. Klapdor-Kleingrothaus, M. H. Klein, M. Klein, U. Klein, K. Kleinknecht, P. Klimek, A. Klimentov, R. Klingenberg, T. Klingl, T. Klioutchnikova, E.-E. Kluge, P. Kluit, S. Kluth, E. Kneringer, E. B. F. G. Knoops, A. Knue, A. Kobayashi, D. Kobayashi, T. Kobayashi, M. Kobel, M. Kocian, P. Kodys, T. Koffas, E. Koffeman, N. M. Köhler, T. Koi, M. Kolb, I. Koletsou, A. A. Komar, T. Kondo, N. Kondrashova, K. Köneke, A. C. König, T. Kono, R. Konoplich, N. Konstantinidis, R. Kopeliansky, S. Koperny, A. K. Kopp, K. Korcyl, K. Kordas, A. Korn, A. A. Korol, I. Korolkov, E. V. Korolkova, O. Kortner, S. Kortner, T. Kosek, V. V. Kostyukhin, A. Kotwal, A. Koulouris, A. Kourkoumeli-Charalampidi, C. Kourkoumelis, E. Kourlitis, V. Kouskoura, A. B. Kowalewska, R. Kowalewski, T. Z. Kowalski, C. Kozakai, W. Kozanecki, A. S. Kozhin, V. A. Kramarenko, G. Kramberger, D. Krasnopevtsev, M. W. Krasny, A. Krasznahorkay, D. Krauss, J. A. Kremer, J. Kretzschmar, K. Kreutzfeldt, P. Krieger, K. Krizka, K. Kroeninger, H. Kroha, J. Kroll, J. Kroll, J. Kroseberg, J. Krstic, U. Kruchonak, H. Krüger, N. Krumnack, M. C. Kruse, T. Kubota, H. Kucuk, S. Kuday, J. T. Kuechler, S. Kuehn, A. Kugel, F. Kuger, T. Kuhl, V. Kukhtin, R. Kukla, Y. Kulchitsky, S. Kuleshov, Y. P. Kulinich, M. Kuna, T. Kunigo, A. Kupco, T. Kupfer, O. Kuprash, H. Kurashige, L. L. Kurchaninov, Y. A. Kurochkin, M. G. Kurth, E. S. Kuwertz, M. Kuze, J. Kvita, T. Kwan, D. Kyriazopoulos, A. La Rosa, J. L. La Rosa Navarro, L. La Rotonda, F. La Ruffa, C. Lacasta, F. Lacava, J. Lacey, D. P. J. Lack, H. Lacker, D. Lacour, E. Ladygin, R. Lafaye, B. Laforge, T. Lagouri, S. Lai, S. Lammers, W. Lampl, E. Lançon, U. Landgraf, M. P. J. Landon, M. C. Lanfermann, V. S. Lang, J. C. Lange, R. J. Langenberg, A. J. Lankford, F. Lanni, K. Lantzsch, A. Lanza, A. Lapertosa, S. Laplace, J. F. Laporte, T. Lari, F. Lasagni Manghi, M. Lassnig, T. S. Lau, P. Laurelli, W. Lavrijsen, A. T. Law, P. Laycock, T. Lazovich, M. Lazzaroni, B. Le, O. Le Dortz, E. Le Guirriec, E. P. Le Quilleuc, M. LeBlanc, T. LeCompte, F. Ledroit-Guillon, C. A. Lee, G. R. Lee, S. C. Lee, L. Lee, B. Lefebvre, G. Lefebvre, M. Lefebvre, F. Legger, C. Leggett, G. Lehmann Miotto, X. Lei, W. A. Leight, M. A. L. Leite, R. Leitner, D. Lellouch, B. Lemmer, K. J. C. Leney, T. Lenz, B. Lenzi, R. Leone, S. Leone, C. Leonidopoulos, G. Lerner, C. Leroy, R. Les, A. A. J. Lesage, C. G. Lester, M. Levchenko, J. Levêque, D. Levin, L. J. Levinson, M. Levy, D. Lewis, B. Li, Changqiao Li, H. Li, L. Li, Q. Li, Q. Li, S. Li, X. Li, Y. Li, Z. Liang, B. Liberti, A. Liblong, K. Lie, J. Liebal, W. Liebig, A. Limosani, K. Lin, S. C. Lin, T. H. Lin, R. A. Linck, B. E. Lindquist, A. E. Lionti, E. Lipeles, A. Lipniacka, M. Lisovyi, T. M. Liss, A. Lister, A. M. Litke, B. Liu, H. Liu, H. Liu, J. K. K. Liu, J. Liu, J. B. Liu, K. Liu, L. Liu, M. Liu, Y. L. Liu, Y. Liu, M. Livan, A. Lleres, J. Llorente Merino, S. L. Lloyd, C. Y. Lo, F. Lo Sterzo, E. M. Lobodzinska, P. Loch, F. K. Loebinger, A. Loesle, K. M. Loew, T. Lohse, K. Lohwasser, M. Lokajicek, B. A. Long, J. D. Long, R. E. Long, L. Longo, K. A. Looper, J. A. Lopez, I. Lopez Paz, A. Lopez Solis, J. Lorenz, N. Lorenzo Martinez, M. Losada, P. J. Lösel, X. Lou, A. Lounis, J. Love, P. A. Love, H. Lu, N. Lu, Y. J. Lu, H. J. Lubatti, C. Luci, A. Lucotte, C. Luedtke, F. Luehring, W. Lukas, L. Luminari, O. Lundberg, B. Lund-Jensen, M. S. Lutz, P. M. Luzi, D. Lynn, R. Lysak, E. Lytken, F. Lyu, V. Lyubushkin, H. Ma, L. L. Ma, Y. Ma, G. Maccarrone, A. Macchiolo, C. M. Macdonald, B. Maček, J. Machado Miguens, D. Madaffari, R. Madar, W. F. Mader, A. Madsen, J. Maeda, S. Maeland, T. Maeno, A. S. Maevskiy, V. Magerl, C. Maiani, C. Maidantchik, T. Maier, A. Maio, O. Majersky, S. Majewski, Y. Makida, N. Makovec, B. Malaescu, Pa. Malecki, V. P. Maleev, F. Malek, U. Mallik, D. Malon, C. Malone, S. Maltezos, S. Malyukov, J. Mamuzic, G. Mancini, I. Mandić, J. Maneira, L. Manhaes de Andrade Filho, J. Manjarres Ramos, K. H. Mankinen, A. Mann, A. Manousos, B. Mansoulie, J. D. Mansour, R. Mantifel, M. Mantoani, S. Manzoni, L. Mapelli, G. Marceca, L. March, L. Marchese, G. Marchiori, M. Marcisovsky, C. A. Marin Tobon, M. Marjanovic, D. E. Marley, F. Marroquim, S. P. Marsden, Z. Marshall, M. U. F Martensson, S. Marti-Garcia, C. B. Martin, T. A. Martin, V. J. Martin, B. Martin dit Latour, M. Martinez, V. I. Martinez Outschoorn, S. Martin-Haugh, V. S. Martoiu, A. C. Martyniuk, A. Marzin, L. Masetti, T. Mashimo, R. Mashinistov, J. Masik, A. L. Maslennikov, L. H. Mason, L. Massa, P. Mastrandrea, A. Mastroberardino, T. Masubuchi, P. Mättig, J. Maurer, S. J. Maxfield, D. A. Maximov, R. Mazini, I. Maznas, S. M. Mazza, N. C. Mc Fadden, G. Mc Goldrick, S. P. Mc Kee, A. McCarn, R. L. McCarthy, T. G. McCarthy, L. I. McClymont, E. F. McDonald, J. A. Mcfayden, G. Mchedlidze, S. J. McMahon, P. C. McNamara, C. J. McNicol, R. A. McPherson, S. Meehan, T. J. Megy, S. Mehlhase, A. Mehta, T. Meideck, K. Meier, B. Meirose, D. Melini, B. R. Mellado Garcia, J. D. Mellenthin, M. Melo, F. Meloni, A. Melzer, S. B. Menary, L. Meng, X. T. Meng, A. Mengarelli, S. Menke, E. Meoni, S. Mergelmeyer, C. Merlassino, P. Mermod, L. Merola, C. Meroni, F. S. Merritt, A. Messina, J. Metcalfe, A. S. Mete, C. Meyer, J-P. Meyer, J. Meyer, H. Meyer Zu Theenhausen, F. Miano, R. P. Middleton, S. Miglioranzi, L. Mijović, G. Mikenberg, M. Mikestikova, M. Mikuž, M. Milesi, A. Milic, D. A. Millar, D. W. Miller, C. Mills, A. Milov, D. A. Milstead, A. A. Minaenko, Y. Minami, I. A. Minashvili, A. I. Mincer, B. Mindur, M. Mineev, Y. Minegishi, Y. Ming, L. M. Mir, A. Mirto, K. P. Mistry, T. Mitani, J. Mitrevski, V. A. Mitsou, A. Miucci, P. S. Miyagawa, A. Mizukami, J. U. Mjörnmark, T. Mkrtchyan, M. Mlynarikova, T. Moa, K. Mochizuki, P. Mogg, S. Mohapatra, S. Molander, R. Moles-Valls, M. C. Mondragon, K. Mönig, J. Monk, E. Monnier, A. Montalbano, J. Montejo Berlingen, F. Monticelli, S. Monzani, R. W. Moore, N. Morange, D. Moreno, M. Moreno Llácer, P. Morettini, S. Morgenstern, D. Mori, T. Mori, M. Morii, M. Morinaga, V. Morisbak, A. K. Morley, G. Mornacchi, J. D. Morris, L. Morvaj, P. Moschovakos, M. Mosidze, H. J. Moss, J. Moss, K. Motohashi, R. Mount, E. Mountricha, E. J. W. Moyse, S. Muanza, F. Mueller, J. Mueller, R. S. P. Mueller, D. Muenstermann, P. Mullen, G. A. Mullier, F. J. Munoz Sanchez, W. J. Murray, H. Musheghyan, M. Muškinja, A. G. Myagkov, M. Myska, B. P. Nachman, O. Nackenhorst, K. Nagai, R. Nagai, K. Nagano, Y. Nagasaka, K. Nagata, M. Nagel, E. Nagy, A. M. Nairz, Y. Nakahama, K. Nakamura, T. Nakamura, I. Nakano, R. F. Naranjo Garcia, R. Narayan, D. I. Narrias Villar, I. Naryshkin, T. Naumann, G. Navarro, R. Nayyar, H. A. Neal, P. Yu. Nechaeva, T. J. Neep, A. Negri, M. Negrini, S. Nektarijevic, C. Nellist, A. Nelson, M. E. Nelson, S. Nemecek, P. Nemethy, M. Nessi, M. S. Neubauer, M. Neumann, P. R. Newman, T. Y. Ng, T. Nguyen Manh, R. B. Nickerson, R. Nicolaidou, J. Nielsen, N. Nikiforou, V. Nikolaenko, I. Nikolic-Audit, K. Nikolopoulos, J. K. Nilsen, P. Nilsson, Y. Ninomiya, A. Nisati, N. Nishu, R. Nisius, I. Nitsche, T. Nitta, T. Nobe, Y. Noguchi, M. Nomachi, I. Nomidis, M. A. Nomura, T. Nooney, M. Nordberg, N. Norjoharuddeen, O. Novgorodova, M. Nozaki, L. Nozka, K. Ntekas, E. Nurse, F. Nuti, K. O’connor, D. C. O’Neil, A. A. O’Rourke, V. O’Shea, F. G. Oakham, H. Oberlack, T. Obermann, J. Ocariz, A. Ochi, I. Ochoa, J. P. Ochoa-Ricoux, S. Oda, S. Odaka, A. Oh, S. H. Oh, C. C. Ohm, H. Ohman, H. Oide, H. Okawa, Y. Okumura, T. Okuyama, A. Olariu, L. F. Oleiro Seabra, S. A. Olivares Pino, D. Oliveira Damazio, A. Olszewski, J. Olszowska, A. Onofre, K. Onogi, P. U. E. Onyisi, H. Oppen, M. J. Oreglia, Y. Oren, D. Orestano, N. Orlando, R. S. Orr, B. Osculati, R. Ospanov, G. Otero y Garzon, H. Otono, M. Ouchrif, F. Ould-Saada, A. Ouraou, K. P. Oussoren, Q. Ouyang, M. Owen, R. E. Owen, V. E. Ozcan, N. Ozturk, K. Pachal, A. Pacheco Pages, L. Pacheco Rodriguez, C. Padilla Aranda, S. Pagan Griso, M. Paganini, F. Paige, G. Palacino, S. Palazzo, S. Palestini, M. Palka, D. Pallin, E. St. Panagiotopoulou, I. Panagoulias, C. E. Pandini, J. G. Panduro Vazquez, P. Pani, S. Panitkin, D. Pantea, L. Paolozzi, Th. D. Papadopoulou, K. Papageorgiou, A. Paramonov, D. Paredes Hernandez, A. J. Parker, M. A. Parker, K. A. Parker, F. Parodi, J. A. Parsons, U. Parzefall, V. R. Pascuzzi, J. M. Pasner, E. Pasqualucci, S. Passaggio, Fr. Pastore, S. Pataraia, J. R. Pater, T. Pauly, B. Pearson, S. Pedraza Lopez, R. Pedro, S. V. Peleganchuk, O. Penc, C. Peng, H. Peng, J. Penwell, B. S. Peralva, M. M. Perego, D. V. Perepelitsa, F. Peri, L. Perini, H. Pernegger, S. Perrella, R. Peschke, V. D. Peshekhonov, K. Peters, R. F. Y. Peters, B. A. Petersen, T. C. Petersen, E. Petit, A. Petridis, C. Petridou, P. Petroff, E. Petrolo, M. Petrov, F. Petrucci, N. E. Pettersson, A. Peyaud, R. Pezoa, F. H. Phillips, P. W. Phillips, G. Piacquadio, E. Pianori, A. Picazio, E. Piccaro, M. A. Pickering, R. Piegaia, J. E. Pilcher, A. D. Pilkington, M. Pinamonti, J. L. Pinfold, H. Pirumov, M. Pitt, L. Plazak, M.-A. Pleier, V. Pleskot, E. Plotnikova, D. Pluth, P. Podberezko, R. Poettgen, R. Poggi, L. Poggioli, I. Pogrebnyak, D. Pohl, I. Pokharel, G. Polesello, A. Poley, A. Policicchio, R. Polifka, A. Polini, C. S. Pollard, V. Polychronakos, K. Pommès, D. Ponomarenko, L. Pontecorvo, G. A. Popeneciu, D. M. Portillo Quintero, S. Pospisil, K. Potamianos, I. N. Potrap, C. J. Potter, H. Potti, T. Poulsen, J. Poveda, M. E. Pozo Astigarraga, P. Pralavorio, A. Pranko, S. Prell, D. Price, M. Primavera, S. Prince, N. Proklova, K. Prokofiev, F. Prokoshin, S. Protopopescu, J. Proudfoot, M. Przybycien, A. Puri, P. Puzo, J. Qian, G. Qin, Y. Qin, A. Quadt, M. Queitsch-Maitland, D. Quilty, S. Raddum, V. Radeka, V. Radescu, S. K. Radhakrishnan, P. Radloff, P. Rados, F. Ragusa, G. Rahal, J. A. Raine, S. Rajagopalan, C. Rangel-Smith, T. Rashid, S. Raspopov, M. G. Ratti, D. M. Rauch, F. Rauscher, S. Rave, I. Ravinovich, J. H. Rawling, M. Raymond, A. L. Read, N. P. Readioff, M. Reale, D. M. Rebuzzi, A. Redelbach, G. Redlinger, R. Reece, R. G. Reed, K. Reeves, L. Rehnisch, J. Reichert, A. Reiss, C. Rembser, H. Ren, M. Rescigno, S. Resconi, E. D. Resseguie, S. Rettie, E. Reynolds, O. L. Rezanova, P. Reznicek, R. Rezvani, R. Richter, S. Richter, E. Richter-Was, O. Ricken, M. Ridel, P. Rieck, C. J. Riegel, J. Rieger, O. Rifki, M. Rijssenbeek, A. Rimoldi, M. Rimoldi, L. Rinaldi, G. Ripellino, B. Ristić, E. Ritsch, I. Riu, F. Rizatdinova, E. Rizvi, C. Rizzi, R. T. Roberts, S. H. Robertson, A. Robichaud-Veronneau, D. Robinson, J. E. M. Robinson, A. Robson, E. Rocco, C. Roda, Y. Rodina, S. Rodriguez Bosca, A. Rodriguez Perez, D. Rodriguez Rodriguez, S. Roe, C. S. Rogan, O. Røhne, J. Roloff, A. Romaniouk, M. Romano, S. M. Romano Saez, E. Romero Adam, N. Rompotis, M. Ronzani, L. Roos, S. Rosati, K. Rosbach, P. Rose, N.-A. Rosien, E. Rossi, L. P. Rossi, J. H. N. Rosten, R. Rosten, M. Rotaru, J. Rothberg, D. Rousseau, A. Rozanov, Y. Rozen, X. Ruan, F. Rubbo, F. Rühr, A. Ruiz-Martinez, Z. Rurikova, N. A. Rusakovich, H. L. Russell, J. P. Rutherfoord, N. Ruthmann, Y. F. Ryabov, M. Rybar, G. Rybkin, S. Ryu, A. Ryzhov, G. F. Rzehorz, A. F. Saavedra, G. Sabato, S. Sacerdoti, H. F-W. Sadrozinski, R. Sadykov, F. Safai Tehrani, P. Saha, M. Sahinsoy, M. Saimpert, M. Saito, T. Saito, H. Sakamoto, Y. Sakurai, G. Salamanna, J. E. Salazar Loyola, D. Salek, P. H. Sales De Bruin, D. Salihagic, A. Salnikov, J. Salt, D. Salvatore, F. Salvatore, A. Salvucci, A. Salzburger, D. Sammel, D. Sampsonidis, D. Sampsonidou, J. Sánchez, V. Sanchez Martinez, A. Sanchez Pineda, H. Sandaker, R. L. Sandbach, C. O. Sander, M. Sandhoff, C. Sandoval, D. P. C. Sankey, M. Sannino, Y. Sano, A. Sansoni, C. Santoni, H. Santos, I. Santoyo Castillo, A. Sapronov, J. G. Saraiva, B. Sarrazin, O. Sasaki, K. Sato, E. Sauvan, G. Savage, P. Savard, N. Savic, C. Sawyer, L. Sawyer, J. Saxon, C. Sbarra, A. Sbrizzi, T. Scanlon, D. A. Scannicchio, J. Schaarschmidt, P. Schacht, B. M. Schachtner, D. Schaefer, L. Schaefer, R. Schaefer, J. Schaeffer, S. Schaepe, S. Schaetzel, U. Schäfer, A. C. Schaffer, D. Schaile, R. D. Schamberger, V. A. Schegelsky, D. Scheirich, M. Schernau, C. Schiavi, S. Schier, L. K. Schildgen, C. Schillo, M. Schioppa, S. Schlenker, K. R. Schmidt-Sommerfeld, K. Schmieden, C. Schmitt, S. Schmitt, S. Schmitz, U. Schnoor, L. Schoeffel, A. Schoening, B. D. Schoenrock, E. Schopf, M. Schott, J. F. P. Schouwenberg, J. Schovancova, S. Schramm, N. Schuh, A. Schulte, M. J. Schultens, H.-C. Schultz-Coulon, H. Schulz, M. Schumacher, B. A. Schumm, Ph. Schune, A. Schwartzman, T. A. Schwarz, H. Schweiger, Ph. Schwemling, R. Schwienhorst, J. Schwindling, A. Sciandra, G. Sciolla, M. Scornajenghi, F. Scuri, F. Scutti, J. Searcy, P. Seema, S. C. Seidel, A. Seiden, J. M. Seixas, G. Sekhniaidze, K. Sekhon, S. J. Sekula, N. Semprini-Cesari, S. Senkin, C. Serfon, L. Serin, L. Serkin, M. Sessa, R. Seuster, H. Severini, T. Sfiligoj, F. Sforza, A. Sfyrla, E. Shabalina, N. W. Shaikh, L. Y. Shan, R. Shang, J. T. Shank, M. Shapiro, P. B. Shatalov, K. Shaw, S. M. Shaw, A. Shcherbakova, C. Y. Shehu, Y. Shen, N. Sherafati, P. Sherwood, L. Shi, S. Shimizu, C. O. Shimmin, M. Shimojima, I. P. J. Shipsey, S. Shirabe, M. Shiyakova, J. Shlomi, A. Shmeleva, D. Shoaleh Saadi, M. J. Shochet, S. Shojaii, D. R. Shope, S. Shrestha, E. Shulga, M. A. Shupe, P. Sicho, A. M. Sickles, P. E. Sidebo, E. Sideras Haddad, O. Sidiropoulou, A. Sidoti, F. Siegert, Dj. Sijacki, J. Silva, S. B. Silverstein, V. Simak, Lj. Simic, S. Simion, E. Simioni, B. Simmons, M. Simon, P. Sinervo, N. B. Sinev, M. Sioli, G. Siragusa, I. Siral, S. Yu. Sivoklokov, J. Sjölin, M. B. Skinner, P. Skubic, M. Slater, T. Slavicek, M. Slawinska, K. Sliwa, R. Slovak, V. Smakhtin, B. H. Smart, J. Smiesko, N. Smirnov, S. Yu. Smirnov, Y. Smirnov, L. N. Smirnova, O. Smirnova, J. W. Smith, M. N. K. Smith, R. W. Smith, M. Smizanska, K. Smolek, A. A. Snesarev, I. M. Snyder, S. Snyder, R. Sobie, F. Socher, A. Soffer, A. Søgaard, D. A. Soh, G. Sokhrannyi, C. A. Solans Sanchez, M. Solar, E. Yu. Soldatov, U. Soldevila, A. A. Solodkov, A. Soloshenko, O. V. Solovyanov, V. Solovyev, P. Sommer, H. Son, A. Sopczak, D. Sosa, C. L. Sotiropoulou, S. Sottocornola, R. Soualah, A. M. Soukharev, D. South, B. C. Sowden, S. Spagnolo, M. Spalla, M. Spangenberg, F. Spanò, D. Sperlich, F. Spettel, T. M. Spieker, R. Spighi, G. Spigo, L. A. Spiller, M. Spousta, R. D. St. Denis, A. Stabile, R. Stamen, S. Stamm, E. Stanecka, R. W. Stanek, C. Stanescu, M. M. Stanitzki, B. S. Stapf, S. Stapnes, E. A. Starchenko, G. H. Stark, J. Stark, S. H Stark, P. Staroba, P. Starovoitov, S. Stärz, R. Staszewski, M. Stegler, P. Steinberg, B. Stelzer, H. J. Stelzer, O. Stelzer-Chilton, H. Stenzel, G. A. Stewart, M. C. Stockton, M. Stoebe, G. Stoicea, P. Stolte, S. Stonjek, A. R. Stradling, A. Straessner, M. E. Stramaglia, J. Strandberg, S. Strandberg, M. Strauss, P. Strizenec, R. Ströhmer, D. M. Strom, R. Stroynowski, A. Strubig, S. A. Stucci, B. Stugu, N. A. Styles, D. Su, J. Su, S. Suchek, Y. Sugaya, M. Suk, V. V. Sulin, DMS Sultan, S. Sultansoy, T. Sumida, S. Sun, X. Sun, K. Suruliz, C. J. E. Suster, M. R. Sutton, S. Suzuki, M. Svatos, M. Swiatlowski, S. P. Swift, I. Sykora, T. Sykora, D. Ta, K. Tackmann, J. Taenzer, A. Taffard, R. Tafirout, E. Tahirovic, N. Taiblum, H. Takai, R. Takashima, E. H. Takasugi, K. Takeda, T. Takeshita, Y. Takubo, M. Talby, A. A. Talyshev, J. Tanaka, M. Tanaka, R. Tanaka, S. Tanaka, R. Tanioka, B. B. Tannenwald, S. Tapia Araya, S. Tapprogge, S. Tarem, G. F. Tartarelli, P. Tas, M. Tasevsky, T. Tashiro, E. Tassi, A. Tavares Delgado, Y. Tayalati, A. C. Taylor, A. J. Taylor, G. N. Taylor, P. T. E. Taylor, W. Taylor, P. Teixeira-Dias, D. Temple, H. Ten Kate, P. K. Teng, J. J. Teoh, F. Tepel, S. Terada, K. Terashi, J. Terron, S. Terzo, M. Testa, R. J. Teuscher, S. J. Thais, T. Theveneaux-Pelzer, F. Thiele, J. P. Thomas, J. Thomas-Wilsker, P. D. Thompson, A. S. Thompson, L. A. Thomsen, E. Thomson, Y. Tian, M. J. Tibbetts, R. E. Ticse Torres, V. O. Tikhomirov, Yu. A. Tikhonov, S. Timoshenko, P. Tipton, S. Tisserant, K. Todome, S. Todorova-Nova, S. Todt, J. Tojo, S. Tokár, K. Tokushuku, E. Tolley, L. Tomlinson, M. Tomoto, L. Tompkins, K. Toms, B. Tong, P. Tornambe, E. Torrence, H. Torres, E. Torró Pastor, J. Toth, F. Touchard, D. R. Tovey, C. J. Treado, T. Trefzger, F. Tresoldi, A. Tricoli, I. M. Trigger, S. Trincaz-Duvoid, M. F. Tripiana, W. Trischuk, B. Trocmé, A. Trofymov, C. Troncon, M. Trottier-McDonald, M. Trovatelli, L. Truong, M. Trzebinski, A. Trzupek, K. W. Tsang, J. C-L. Tseng, P. V. Tsiareshka, G. Tsipolitis, N. Tsirintanis, S. Tsiskaridze, V. Tsiskaridze, E. G. Tskhadadze, I. I. Tsukerman, V. Tsulaia, S. Tsuno, D. Tsybychev, Y. Tu, A. Tudorache, V. Tudorache, T. T. Tulbure, A. N. Tuna, S. Turchikhin, D. Turgeman, I. Turk Cakir, R. Turra, P. M. Tuts, G. Ucchielli, I. Ueda, M. Ughetto, F. Ukegawa, G. Unal, A. Undrus, G. Unel, F. C. Ungaro, Y. Unno, C. Unverdorben, J. Urban, P. Urquijo, P. Urrejola, G. Usai, J. Usui, L. Vacavant, V. Vacek, B. Vachon, K. O. H. Vadla, A. Vaidya, C. Valderanis, E. Valdes Santurio, M. Valente, S. Valentinetti, A. Valero, L. Valéry, S. Valkar, A. Vallier, J. A. Valls Ferrer, W. Van Den Wollenberg, H. van der Graaf, P. van Gemmeren, J. Van Nieuwkoop, I. van Vulpen, M. C. van Woerden, M. Vanadia, W. Vandelli, A. Vaniachine, P. Vankov, G. Vardanyan, R. Vari, E. W. Varnes, C. Varni, T. Varol, D. Varouchas, A. Vartapetian, K. E. Varvell, J. G. Vasquez, G. A. Vasquez, F. Vazeille, D. Vazquez Furelos, T. Vazquez Schroeder, J. Veatch, V. Veeraraghavan, L. M. Veloce, F. Veloso, S. Veneziano, A. Ventura, M. Venturi, N. Venturi, A. Venturini, V. Vercesi, M. Verducci, W. Verkerke, A. T. Vermeulen, J. C. Vermeulen, M. C. Vetterli, N. Viaux Maira, O. Viazlo, I. Vichou, T. Vickey, O. E. Vickey Boeriu, G. H. A. Viehhauser, S. Viel, L. Vigani, M. Villa, M. Villaplana Perez, E. Vilucchi, M. G. Vincter, V. B. Vinogradov, A. Vishwakarma, C. Vittori, I. Vivarelli, S. Vlachos, M. Vogel, P. Vokac, G. Volpi, H. von der Schmitt, E. von Toerne, V. Vorobel, K. Vorobev, M. Vos, R. Voss, J. H. Vossebeld, N. Vranjes, M. Vranjes Milosavljevic, V. Vrba, M. Vreeswijk, R. Vuillermet, I. Vukotic, P. Wagner, W. Wagner, J. Wagner-Kuhr, H. Wahlberg, S. Wahrmund, J. Walder, R. Walker, W. Walkowiak, V. Wallangen, C. Wang, C. Wang, F. Wang, H. Wang, H. Wang, J. Wang, J. Wang, Q. Wang, R.-J. Wang, R. Wang, S. M. Wang, T. Wang, W. Wang, W. Wang, Z. Wang, C. Wanotayaroj, A. Warburton, C. P. Ward, D. R. Wardrope, A. Washbrook, P. M. Watkins, A. T. Watson, M. F. Watson, G. Watts, S. Watts, B. M. Waugh, A. F. Webb, S. Webb, M. S. Weber, S. M. Weber, S. W. Weber, S. A. Weber, J. S. Webster, A. R. Weidberg, B. Weinert, J. Weingarten, M. Weirich, C. Weiser, H. Weits, P. S. Wells, T. Wenaus, T. Wengler, S. Wenig, N. Wermes, M. D. Werner, P. Werner, M. Wessels, T. D. Weston, K. Whalen, N. L. Whallon, A. M. Wharton, A. S. White, A. White, M. J. White, R. White, D. Whiteson, B. W. Whitmore, F. J. Wickens, W. Wiedenmann, M. Wielers, C. Wiglesworth, L. A. M. Wiik-Fuchs, A. Wildauer, F. Wilk, H. G. Wilkens, H. H. Williams, S. Williams, C. Willis, S. Willocq, J. A. Wilson, I. Wingerter-Seez, E. Winkels, F. Winklmeier, O. J. Winston, B. T. Winter, M. Wittgen, M. Wobisch, T. M. H. Wolf, R. Wolff, M. W. Wolter, H. Wolters, V. W. S. Wong, N. L. Woods, S. D. Worm, B. K. Wosiek, J. Wotschack, K. W. Wozniak, M. Wu, S. L. Wu, X. Wu, Y. Wu, T. R. Wyatt, B. M. Wynne, S. Xella, Z. Xi, L. Xia, D. Xu, L. Xu, T. Xu, B. Yabsley, S. Yacoob, D. Yamaguchi, Y. Yamaguchi, A. Yamamoto, S. Yamamoto, T. Yamanaka, F. Yamane, M. Yamatani, Y. Yamazaki, Z. Yan, H. Yang, H. Yang, Y. Yang, Z. Yang, W-M. Yao, Y. C. Yap, Y. Yasu, E. Yatsenko, K. H. Yau Wong, J. Ye, S. Ye, I. Yeletskikh, E. Yigitbasi, E. Yildirim, K. Yorita, K. Yoshihara, C. Young, C. J. S. Young, J. Yu, J. Yu, S. P. Y. Yuen, I. Yusuff, B. Zabinski, G. Zacharis, R. Zaidan, A. M. Zaitsev, N. Zakharchuk, J. Zalieckas, A. Zaman, S. Zambito, D. Zanzi, C. Zeitnitz, G. Zemaityte, A. Zemla, J. C. Zeng, Q. Zeng, O. Zenin, T. Ženiš, D. Zerwas, D. Zhang, D. Zhang, F. Zhang, G. Zhang, H. Zhang, J. Zhang, L. Zhang, L. Zhang, M. Zhang, P. Zhang, R. Zhang, R. Zhang, X. Zhang, Y. Zhang, Z. Zhang, X. Zhao, Y. Zhao, Z. Zhao, A. Zhemchugov, B. Zhou, C. Zhou, L. Zhou, M. Zhou, M. Zhou, N. Zhou, C. G. Zhu, H. Zhu, J. Zhu, Y. Zhu, X. Zhuang, K. Zhukov, A. Zibell, D. Zieminska, N. I. Zimine, C. Zimmermann, S. Zimmermann, Z. Zinonos, M. Zinser, M. Ziolkowski, L. Živković, G. Zobernig, A. Zoccoli, R. Zou, M. zur Nedden, L. Zwalinski, ATLAS Collaboration

**Affiliations:** 1 Department of Physics, University of Adelaide, Adelaide, Australia; 2 Physics Department, SUNY Albany, Albany, NY USA; 3 Department of Physics, University of Alberta, Edmonton, AB Canada; 4 Department of Physics, Ankara University, Ankara, Turkey; 5 Istanbul Aydin University, Istanbul, Turkey; 6 Division of Physics, TOBB University of Economics and Technology, Ankara, Turkey; 7 LAPP, CNRS/IN2P3 and Université Savoie Mont Blanc, Annecy-le-Vieux, France; 8 High Energy Physics Division, Argonne National Laboratory, Argonne, IL USA; 9 Department of Physics, University of Arizona, Tucson, AZ USA; 10 Department of Physics, The University of Texas at Arlington, Arlington, TX USA; 11 Physics Department, National and Kapodistrian University of Athens, Athens, Greece; 12 Physics Department, National Technical University of Athens, Zografou, Greece; 13 Department of Physics, The University of Texas at Austin, Austin, TX USA; 14 Institute of Physics, Azerbaijan Academy of Sciences, Baku, Azerbaijan; 15 Institut de Física d’Altes Energies (IFAE), The Barcelona Institute of Science and Technology, Barcelona, Spain; 16 Institute of Physics, University of Belgrade, Belgrade, Serbia; 17 Department for Physics and Technology, University of Bergen, Bergen, Norway; 18 Physics Division, Lawrence Berkeley National Laboratory, University of California, Berkeley, CA USA; 19 Department of Physics, Humboldt University, Berlin, Germany; 20 Albert Einstein Center for Fundamental Physics, Laboratory for High Energy Physics, University of Bern, Bern, Switzerland; 21 School of Physics and Astronomy, University of Birmingham, Birmingham, UK; 22 Department of Physics, Bogazici University, Istanbul, Turkey; 23 Department of Physics Engineering, Gaziantep University, Gaziantep, Turkey; 24 Faculty of Engineering and Natural Sciences, Istanbul Bilgi University, Istanbul, Turkey; 25 Faculty of Engineering and Natural Sciences, Bahcesehir University, Istanbul, Turkey; 26 Centro de Investigaciones, Universidad Antonio Narino, Bogotá, Colombia; 27 INFN Sezione di Bologna, Bologna, Italy; 28 Dipartimento di Fisica e Astronomia, Università di Bologna, Bologna, Italy; 29 Physikalisches Institut, University of Bonn, Bonn, Germany; 30 Department of Physics, Boston University, Boston, MA USA; 31 Department of Physics, Brandeis University, Waltham, MA USA; 32 Universidade Federal do Rio De Janeiro COPPE/EE/IF, Rio de Janeiro, Brazil; 33 Electrical Circuits Department, Federal University of Juiz de Fora (UFJF), Juiz de Fora, Brazil; 34 Federal University of Sao Joao del Rei (UFSJ), Sao Joao del Rei, Brazil; 35 Instituto de Fisica, Universidade de Sao Paulo, São Paulo, Brazil; 36 Physics Department, Brookhaven National Laboratory, Upton, NY USA; 37 Transilvania University of Brasov, Brasov, Romania; 38 Horia Hulubei National Institute of Physics and Nuclear Engineering, Bucharest, Romania; 39 Department of Physics, Alexandru Ioan Cuza University of Iasi, Iasi, Romania; 40 Physics Department, National Institute for Research and Development of Isotopic and Molecular Technologies, Cluj-Napoca, Romania; 41 University Politehnica Bucharest, Bucharest, Romania; 42 West University in Timisoara, Timisoara, Romania; 43 Departamento de Física, Universidad de Buenos Aires, Buenos Aires, Argentina; 44 Cavendish Laboratory, University of Cambridge, Cambridge, UK; 45 Department of Physics, Carleton University, Ottawa, ON Canada; 46 CERN, Geneva, Switzerland; 47 Enrico Fermi Institute, University of Chicago, Chicago, IL USA; 48 Departamento de Física, Pontificia Universidad Católica de Chile, Santiago, Chile; 49 Departamento de Física, Universidad Técnica Federico Santa María, Valparaiso, Chile; 50 Institute of High Energy Physics, Chinese Academy of Sciences, Beijing, China; 51 Department of Physics, Nanjing University, Nanjing, Jiangsu China; 52 Physics Department, Tsinghua University, Beijing, 100084 China; 53 Department of Modern Physics and State Key Laboratory of Particle Detection and Electronics, University of Science and Technology of China, Hefei, Anhui China; 54 School of Physics, Shandong University, Jinan, Shandong China; 55 Department of Physics and Astronomy, Key Laboratory for Particle Physics, Astrophysics and Cosmology, Ministry of Education, Shanghai Key Laboratory for Particle Physics and Cosmology, Shanghai Jiao Tong University, Shanghai (also at PKU-CHEP), Shanghai, China; 56 Université Clermont Auvergne, CNRS/IN2P3, LPC, Clermont-Ferrand, France; 57 Nevis Laboratory, Columbia University, Irvington, NY USA; 58 Niels Bohr Institute, University of Copenhagen, Copenhagen, Denmark; 59 INFN Gruppo Collegato di Cosenza, Laboratori Nazionali di Frascati, Frascati, Italy; 60 Dipartimento di Fisica, Università della Calabria, Rende, Italy; 61 Faculty of Physics and Applied Computer Science, AGH University of Science and Technology, Kraków, Poland; 62 Marian Smoluchowski Institute of Physics, Jagiellonian University, Kraków, Poland; 63 Institute of Nuclear Physics, Polish Academy of Sciences, Kraków, Poland; 64 Physics Department, Southern Methodist University, Dallas, TX USA; 65 Physics Department, University of Texas at Dallas, Richardson, TX USA; 66 DESY, Hamburg and Zeuthen, Germany; 67 Lehrstuhl für Experimentelle Physik IV, Technische Universität Dortmund, Dortmund, Germany; 68 Institut für Kern- und Teilchenphysik, Technische Universität Dresden, Dresden, Germany; 69 Department of Physics, Duke University, Durham, NC USA; 70 SUPA-School of Physics and Astronomy, University of Edinburgh, Edinburgh, UK; 71 INFN e Laboratori Nazionali di Frascati, Frascati, Italy; 72 Fakultät für Mathematik und Physik, Albert-Ludwigs-Universität, Freiburg, Germany; 73 Departement de Physique Nucleaire et Corpusculaire, Université de Genève, Geneva, Switzerland; 74 INFN Sezione di Genova, Genoa, Italy; 75 Dipartimento di Fisica, Università di Genova, Genoa, Italy; 76 E. Andronikashvili Institute of Physics, Iv. Javakhishvili Tbilisi State University, Tbilisi, Georgia; 77 High Energy Physics Institute, Tbilisi State University, Tbilisi, Georgia; 78 II Physikalisches Institut, Justus-Liebig-Universität Giessen, Giessen, Germany; 79 SUPA-School of Physics and Astronomy, University of Glasgow, Glasgow, UK; 80 II Physikalisches Institut, Georg-August-Universität, Göttingen, Germany; 81 Laboratoire de Physique Subatomique et de Cosmologie, Université Grenoble-Alpes, CNRS/IN2P3, Grenoble, France; 82 Laboratory for Particle Physics and Cosmology, Harvard University, Cambridge, MA USA; 83 Kirchhoff-Institut für Physik, Ruprecht-Karls-Universität Heidelberg, Heidelberg, Germany; 84 Physikalisches Institut, Ruprecht-Karls-Universität Heidelberg, Heidelberg, Germany; 85 Faculty of Applied Information Science, Hiroshima Institute of Technology, Hiroshima, Japan; 86 Department of Physics, The Chinese University of Hong Kong, Shatin, N.T. Hong Kong; 87 Department of Physics, The University of Hong Kong, Hong Kong, China; 88 Department of Physics, Institute for Advanced Study, The Hong Kong University of Science and Technology, Clear Water Bay, Kowloon, Hong Kong, China; 89 Department of Physics, National Tsing Hua University, Taiwan, Taiwan; 90 Department of Physics, Indiana University, Bloomington, IN USA; 91 Institut für Astro- und Teilchenphysik, Leopold-Franzens-Universität, Innsbruck, Austria; 92 University of Iowa, Iowa City, IA USA; 93 Department of Physics and Astronomy, Iowa State University, Ames, IA USA; 94 Joint Institute for Nuclear Research, JINR Dubna, Dubna, Russia; 95 KEK, High Energy Accelerator Research Organization, Tsukuba, Japan; 96 Graduate School of Science, Kobe University, Kobe, Japan; 97 Faculty of Science, Kyoto University, Kyoto, Japan; 98 Kyoto University of Education, Kyoto, Japan; 99 Research Center for Advanced Particle Physics and Department of Physics, Kyushu University, Fukuoka, Japan; 100 Instituto de Física La Plata, Universidad Nacional de La Plata and CONICET, La Plata, Argentina; 101 Physics Department, Lancaster University, Lancaster, UK; 102 INFN Sezione di Lecce, Lecce, Italy; 103 Dipartimento di Matematica e Fisica, Università del Salento, Lecce, Italy; 104 Oliver Lodge Laboratory, University of Liverpool, Liverpool, UK; 105 Department of Experimental Particle Physics, Jožef Stefan Institute and Department of Physics, University of Ljubljana, Ljubljana, Slovenia; 106 School of Physics and Astronomy, Queen Mary University of London, London, UK; 107 Department of Physics, Royal Holloway University of London, Surrey, UK; 108 Department of Physics and Astronomy, University College London, London, UK; 109 Louisiana Tech University, Ruston, LA USA; 110 Laboratoire de Physique Nucléaire et de Hautes Energies, UPMC and Université Paris-Diderot and CNRS/IN2P3, Paris, France; 111 Fysiska institutionen, Lunds universitet, Lund, Sweden; 112 Departamento de Fisica Teorica C-15, Universidad Autonoma de Madrid, Madrid, Spain; 113 Institut für Physik, Universität Mainz, Mainz, Germany; 114 School of Physics and Astronomy, University of Manchester, Manchester, UK; 115 CPPM, Aix-Marseille Université and CNRS/IN2P3, Marseille, France; 116 Department of Physics, University of Massachusetts, Amherst, MA USA; 117 Department of Physics, McGill University, Montreal, QC Canada; 118 School of Physics, University of Melbourne, Victoria, Australia; 119 Department of Physics, The University of Michigan, Ann Arbor, MI USA; 120 Department of Physics and Astronomy, Michigan State University, East Lansing, MI USA; 121 INFN Sezione di Milano, Milan, Italy; 122 Dipartimento di Fisica, Università di Milano, Milan, Italy; 123 B.I. Stepanov Institute of Physics, National Academy of Sciences of Belarus, Minsk, Republic of Belarus; 124 Research Institute for Nuclear Problems of Byelorussian State University, Minsk, Republic of Belarus; 125 Group of Particle Physics, University of Montreal, Montreal, QC Canada; 126 P.N. Lebedev Physical Institute of the Russian Academy of Sciences, Moscow, Russia; 127 Institute for Theoretical and Experimental Physics (ITEP), Moscow, Russia; 128 National Research Nuclear University MEPhI, Moscow, Russia; 129 D.V. Skobeltsyn Institute of Nuclear Physics, M.V. Lomonosov Moscow State University, Moscow, Russia; 130 Fakultät für Physik, Ludwig-Maximilians-Universität München, Munich, Germany; 131 Max-Planck-Institut für Physik (Werner-Heisenberg-Institut), Munich, Germany; 132 Nagasaki Institute of Applied Science, Nagasaki, Japan; 133 Graduate School of Science and Kobayashi-Maskawa Institute, Nagoya University, Nagoya, Japan; 134 INFN Sezione di Napoli, Naples, Italy; 135 Dipartimento di Fisica, Università di Napoli, Naples, Italy; 136 Department of Physics and Astronomy, University of New Mexico, Albuquerque, NM USA; 137 Institute for Mathematics, Astrophysics and Particle Physics, Radboud University Nijmegen/Nikhef, Nijmegen, The Netherlands; 138 Nikhef National Institute for Subatomic Physics, University of Amsterdam, Amsterdam, The Netherlands; 139 Department of Physics, Northern Illinois University, DeKalb, IL USA; 140 Budker Institute of Nuclear Physics, SB RAS, Novosibirsk, Russia; 141 Department of Physics, New York University, New York, NY USA; 142 Ohio State University, Columbus, OH USA; 143 Faculty of Science, Okayama University, Okayama, Japan; 144 Homer L. Dodge Department of Physics and Astronomy, University of Oklahoma, Norman, OK USA; 145 Department of Physics, Oklahoma State University, Stillwater, OK USA; 146 Palacký University, RCPTM, Olomouc, Czech Republic; 147 Center for High Energy Physics, University of Oregon, Eugene, OR USA; 148 LAL, Univ. Paris-Sud, CNRS/IN2P3, Université Paris-Saclay, Orsay, France; 149 Graduate School of Science, Osaka University, Osaka, Japan; 150 Department of Physics, University of Oslo, Oslo, Norway; 151 Department of Physics, Oxford University, Oxford, UK; 152 INFN Sezione di Pavia, Pavia, Italy; 153 Dipartimento di Fisica, Università di Pavia, Pavia, Italy; 154 Department of Physics, University of Pennsylvania, Philadelphia, PA USA; 155 National Research Centre “Kurchatov Institute” B.P. Konstantinov Petersburg Nuclear Physics Institute, St. Petersburg, Russia; 156 INFN Sezione di Pisa, Pisa, Italy; 157 Dipartimento di Fisica E. Fermi, Università di Pisa, Pisa, Italy; 158 Department of Physics and Astronomy, University of Pittsburgh, Pittsburgh, PA USA; 159 Laboratório de Instrumentação e Física Experimental de Partículas-LIP, Lisbon, Portugal; 160 Faculdade de Ciências, Universidade de Lisboa, Lisbon, Portugal; 161 Department of Physics, University of Coimbra, Coimbra, Portugal; 162 Centro de Física Nuclear da Universidade de Lisboa, Lisbon, Portugal; 163 Departamento de Fisica, Universidade do Minho, Braga, Portugal; 164 Departamento de Fisica Teorica y del Cosmos, Universidad de Granada, Granada, Spain; 165 Dep Fisica and CEFITEC of Faculdade de Ciencias e Tecnologia, Universidade Nova de Lisboa, Caparica, Portugal; 166 Institute of Physics, Academy of Sciences of the Czech Republic, Prague, Czech Republic; 167 Czech Technical University in Prague, Prague, Czech Republic; 168 Faculty of Mathematics and Physics, Charles University, Prague, Czech Republic; 169 State Research Center Institute for High Energy Physics (Protvino), NRC KI, Protvino, Russia; 170 Particle Physics Department, Rutherford Appleton Laboratory, Didcot, UK; 171 INFN Sezione di Roma, Rome, Italy; 172 Dipartimento di Fisica, Sapienza Università di Roma, Rome, Italy; 173 INFN Sezione di Roma Tor Vergata, Rome, Italy; 174 Dipartimento di Fisica, Università di Roma Tor Vergata, Rome, Italy; 175 INFN Sezione di Roma Tre, Rome, Italy; 176 Dipartimento di Matematica e Fisica, Università Roma Tre, Rome, Italy; 177 Faculté des Sciences Ain Chock, Réseau Universitaire de Physique des Hautes Energies-Université Hassan II, Casablanca, Morocco; 178 Centre National de l’Energie des Sciences Techniques Nucleaires, Rabat, Morocco; 179 Faculté des Sciences Semlalia, Université Cadi Ayyad, LPHEA-Marrakech, Marrakech, Morocco; 180 Faculté des Sciences, Université Mohamed Premier and LPTPM, Oujda, Morocco; 181 Faculté des Sciences, Université Mohammed V, Rabat, Morocco; 182 DSM/IRFU (Institut de Recherches sur les Lois Fondamentales de l’Univers), CEA Saclay (Commissariat à l’Energie Atomique et aux Energies Alternatives), Gif-sur-Yvette, France; 183 Santa Cruz Institute for Particle Physics, University of California Santa Cruz, Santa Cruz, CA USA; 184 Department of Physics, University of Washington, Seattle, WA USA; 185 Department of Physics and Astronomy, University of Sheffield, Sheffield, UK; 186 Department of Physics, Shinshu University, Nagano, Japan; 187 Department Physik, Universität Siegen, Siegen, Germany; 188 Department of Physics, Simon Fraser University, Burnaby, BC Canada; 189 SLAC National Accelerator Laboratory, Stanford, CA USA; 190 Faculty of Mathematics, Physics and Informatics, Comenius University, Bratislava, Slovak Republic; 191 Department of Subnuclear Physics, Institute of Experimental Physics of the Slovak Academy of Sciences, Kosice, Slovak Republic; 192 Department of Physics, University of Cape Town, Cape Town, South Africa; 193 Department of Physics, University of Johannesburg, Johannesburg, South Africa; 194 School of Physics, University of the Witwatersrand, Johannesburg, South Africa; 195 Department of Physics, Stockholm University, Stockholm, Sweden; 196 The Oskar Klein Centre, Stockholm, Sweden; 197 Physics Department, Royal Institute of Technology, Stockholm, Sweden; 198 Departments of Physics and Astronomy and Chemistry, Stony Brook University, Stony Brook, NY USA; 199 Department of Physics and Astronomy, University of Sussex, Brighton, UK; 200 School of Physics, University of Sydney, Sydney, Australia; 201 Institute of Physics, Academia Sinica, Taipei, Taiwan; 202 Department of Physics, Technion: Israel Institute of Technology, Haifa, Israel; 203 Raymond and Beverly Sackler School of Physics and Astronomy, Tel Aviv University, Tel Aviv, Israel; 204 Department of Physics, Aristotle University of Thessaloniki, Thessaloníki, Greece; 205 International Center for Elementary Particle Physics and Department of Physics, The University of Tokyo, Tokyo, Japan; 206 Graduate School of Science and Technology, Tokyo Metropolitan University, Tokyo, Japan; 207 Department of Physics, Tokyo Institute of Technology, Tokyo, Japan; 208 Tomsk State University, Tomsk, Russia; 209 Department of Physics, University of Toronto, Toronto, ON Canada; 210 INFN-TIFPA, Trento, Italy; 211 University of Trento, Trento, Italy; 212 TRIUMF, Vancouver, BC Canada; 213 Department of Physics and Astronomy, York University, Toronto, ON Canada; 214 Faculty of Pure and Applied Sciences, and Center for Integrated Research in Fundamental Science and Engineering, University of Tsukuba, Tsukuba, Japan; 215 Department of Physics and Astronomy, Tufts University, Medford, MA USA; 216 Department of Physics and Astronomy, University of California Irvine, Irvine, CA USA; 217 INFN Gruppo Collegato di Udine, Sezione di Trieste, Udine, Italy; 218 ICTP, Trieste, Italy; 219 Dipartimento di Chimica, Fisica e Ambiente, Università di Udine, Udine, Italy; 220 Department of Physics and Astronomy, University of Uppsala, Uppsala, Sweden; 221 Department of Physics, University of Illinois, Urbana, IL USA; 222 Instituto de Fisica Corpuscular (IFIC), Centro Mixto Universidad de Valencia-CSIC, Valencia, Spain; 223 Department of Physics, University of British Columbia, Vancouver, BC Canada; 224 Department of Physics and Astronomy, University of Victoria, Victoria, BC Canada; 225 Department of Physics, University of Warwick, Coventry, UK; 226 Waseda University, Tokyo, Japan; 227 Department of Particle Physics, The Weizmann Institute of Science, Rehovot, Israel; 228 Department of Physics, University of Wisconsin, Madison, WI USA; 229 Fakultät für Physik und Astronomie, Julius-Maximilians-Universität, Würzburg, Germany; 230 Fakultät für Mathematik und Naturwissenschaften, Fachgruppe Physik, Bergische Universität Wuppertal, Wuppertal, Germany; 231 Department of Physics, Yale University, New Haven, CT USA; 232 Yerevan Physics Institute, Yerevan, Armenia; 233 Centre de Calcul de l’Institut National de Physique Nucléaire et de Physique des Particules (IN2P3), Villeurbanne, France; 234 Academia Sinica Grid Computing, Institute of Physics, Academia Sinica, Taipei, Taiwan; 235 CERN, 1211 Geneva 23, Switzerland

## Abstract

A search is performed for new phenomena in events having a photon with high transverse momentum and a jet collected in $36.7~\text {fb}^{-1}$ of proton–proton collisions at a centre-of-mass energy of $\sqrt{s}$ = 13 TeV recorded with the ATLAS detector at the Large Hadron Collider. The invariant mass distribution of the leading photon and jet is examined to look for the resonant production of new particles or the presence of new high-mass states beyond the Standard Model. No significant deviation from the background-only hypothesis is observed and cross-section limits for generic Gaussian-shaped resonances are extracted. Excited quarks hypothesized in quark compositeness models and high-mass states predicted in quantum black hole models with extra dimensions are also examined in the analysis. The observed data exclude, at 95% confidence level, the mass range below 5.3 TeV for excited quarks and 7.1 TeV (4.4 TeV) for quantum black holes in the Arkani-Hamed–Dimopoulos–Dvali (Randall–Sundrum) model with six (one) extra dimensions.

## Introduction

This paper reports a search for new phenomena in events with a photon and a jet produced from proton–proton ($pp$) collisions at $\sqrt{s}$ = 13 TeV, collected with the ATLAS detector at the Large Hadron Collider (LHC). Prompt photons in association with jets are copiously produced at the LHC, mainly through quark–gluon scattering ($qg \rightarrow q\gamma$). The $\gamma$ + jet(s) final state provides a sensitive probe for a class of phenomena beyond the Standard Model (SM) that could manifest themselves in the high invariant mass ($m_{\gamma j}$) region of the $\gamma$ + jet system. The search is performed by looking for localized excesses of events in the $m_{\gamma j}$ distribution with respect to the SM prediction. Two classes of benchmark signal models are considered.

The first class of benchmark models is based on a generic Gaussian-shaped mass distribution with different values of its mean and standard deviation. This provides a generic interpretation for the presence of signals with different Gaussian widths, ranging from a resonance with a width similar to the reconstructed $$m_{\gamma j}$$ resolution of $$\sim 2$$% to wide resonances with a width up to 15%. The second class of benchmark models is based on signals beyond the SM that are implemented in Monte Carlo (MC) simulation and appear as broad peaks in the $$m_{\gamma j}$$ spectrum. This paper considers two scenarios for physics beyond the SM: quarks as composite particles and extra spatial dimensions. In the first case, if quarks are composed of more fundamental constituents bound together by some unknown interaction, new effects should appear depending on the value of the compositeness scale $$\Lambda $$. In particular, if $$\Lambda $$ is sufficiently smaller than the centre-of-mass energy, excited quark ($$q^*$$) states may be produced in high-energy $$pp$$ collisions at the LHC  [1–3]. The $$q^*$$ production at the LHC could result in a resonant peak at the mass of the $$q^*$$ ($$m_{q^*}$$) in the $$m_{\gamma j}$$ distribution if the $$q^*$$ can decay into a photon and a quark ($$qg \rightarrow q^* \rightarrow q\gamma $$). In the present search, only the SM gauge interactions are considered for $$q^*$$ production. In the second scenario, the existence of extra spatial dimensions (EDs) is assumed to provide a solution to the hierarchy problem [4–6]. Certain types of ED models predict the fundamental Planck scale $$M^*$$ in the $$4+n$$ dimensions (*n* being the number of extra spatial dimensions) to be at the TeV scale, and thus accessible in $$pp$$ collisions at $$\sqrt{s}$$ = 13 TeV at the LHC. In such a TeV-scale $$M^*$$ scenario of the extra dimensions, quantum black holes (QBHs) may be produced at the LHC as a continuum above the threshold mass ($$M_{\text {th}}$$) and then decay into a small number of final-state particles including photon–quark/gluon pairs before they are able to thermalize [7–10]. In this case a broad resonance-like structure could be observed just above $$M_{\text {th}}$$ on top of the SM $$m_{\gamma j}$$ distribution. The $$M_{\text {th}}$$ value for QBH production is taken to be equal to $$M^*$$ while the maximum allowed QBH mass is set to either $$3M^* $$ or the LHC $$pp$$ centre-of-mass energy of 13 TeV, whichever is smaller. The upper bound on the mass ensures that the QBH production is far from the “thermal” regime, where the classical description of the black hole and its decay into high-multiplicity final states should be used. In this paper, the extra-dimensions model proposed by Arkani-Hamed, Dimopoulous and Dvali (ADD) [11] with $$n=6$$ flat EDs and the one by Randall and Sundrum (RS1) [12] with $$n=1$$ warped ED are considered.

The ATLAS and CMS experiments at the LHC have performed searches for excited quarks in the $\gamma +\text {jet}$ final state using $pp$ collision data recorded at $\sqrt{s}$ = 7 TeV [13], 8 TeV [14, 15] and 13 TeV [16]. In the ATLAS searches, limits for generic Gaussian-shaped resonances were obtained at 7, 8 and 13 TeV while a limit for QBHs in the ADD model ($n=6$) was first obtained at 8 TeV. The ATLAS search at 13 TeV with data taken in 2015 was further extended to constrain QBHs in the RS1 model ($n=1$). No significant excess of events was observed in any of these searches, and the lower mass limits of 4.4 TeV for the $q^*$ and 6.2 (3.8) TeV for QBHs in the ADD (RS1) model were set, currently representing the most stringent limits for the decay into a photon and a jet. For a Gaussian-shaped resonance a cross-section upper limit of 0.8 (1.0) fb at $\sqrt{s}$ = 13 TeV was obtained, for example, for a mass of 5 TeV and a width of 2% (15%).

The dijet resonance searches at ATLAS [17, 18] and CMS [19] using $pp$ collisions at $\sqrt{s}$ = 13 TeV also set limits on the production cross-sections of excited quarks and QBHs. The search in the $\gamma +\text {jet}$ final state presented here complements the dijet results and provides an independent check for the presence of these signals in different decay channels.

This paper presents the search based on the full 2015 and 2016 data set recorded with the ATLAS detector, corresponding to $36.7~\text {fb}^{-1}$ of $pp$ collisions at $\sqrt{s}$ = 13 TeV. The analysis strategy is unchanged from the one reported in Ref. [16], focusing on the region where the $\gamma$ + jet system has a high invariant mass.

The paper is organized as follows. In Sect. 2 a brief description of the ATLAS detector is given. Section 3 summarizes the data and simulation samples used in this study. The event selection is discussed in Sect. 4. The signal and background modelling are presented in Sect. 5 together with the signal search and limit-setting strategies. Finally the results are discussed in Sect. 6 and the conclusions are given in Sect. 7.

## ATLAS detector

The ATLAS detector at the LHC is a multi-purpose, forward-backward symmetric detector^1^ with almost full solid angle coverage, and is described in detail elsewhere [20, 21]. Most relevant for this analysis are the inner detector (ID) and the calorimeter system composed of electromagnetic (EM) and hadronic calorimeters. The ID consists of a silicon pixel detector, a silicon microstrip tracker and a transition radiation tracker, all immersed in a 2 T axial magnetic field, and provides charged-particle tracking in the range $|\eta |<2.5$. The electromagnetic calorimeter is a lead/liquid-argon (LAr) sampling calorimeter with accordion geometry. The calorimeter is divided into a barrel section covering $|\eta |<1.475$ and two endcap sections covering $1.375<|\eta |<3.2$. For $|\eta |<2.5$ it is divided into three layers in depth, which are finely segmented in $\eta$ and $\phi$. In the region $|\eta |<1.8$, an additional thin LAr presampler layer is used to correct for fluctuations in the energy losses in the material upstream of the calorimeters. The hadronic calorimeter is a sampling calorimeter composed of steel/scintillator tiles in the central region ($|\eta |<1.7$), while copper/LAr modules are used in the endcap ($1.5<|\eta |<3.2$) regions. The forward regions ($3.1<|\eta |<4.9$) are instrumented with copper/LAr and tungsten/LAr calorimeter modules optimized for electromagnetic and hadronic measurements, respectively. Surrounding the calorimeters is a muon spectrometer that includes three air-core superconducting toroidal magnets and multiple types of tracking chambers, providing precision tracking for muons within $|\eta |<2.7$ and trigger capability within $|\eta |<2.4$.

A dedicated two-level trigger system is used for the online event selection [22]. Events are selected using a first-level trigger implemented in custom electronics, which reduces the event rate to a design value of 100 kHz using a subset of the detector information. This is followed by a software-based trigger that reduces the accepted event rate to 1 kHz on average by refining the first-level trigger selection.

## Data and Monte Carlo simulations

The data sample used in this analysis was collected from $pp$ collisions in the 2015–2016 LHC run at a centre-of-mass energy of 13 TeV, and corresponds to an integrated luminosity of $36.7\pm 1.2~\text {fb}^{-1}$. The uncertainty was derived, following a methodology similar to that detailed in Ref. [23], from a preliminary calibration of the luminosity scale using *x*–*y* beam-separation scans performed in August 2015 and May 2016. The data are required to satisfy a number of quality criteria ensuring that the relevant detectors were operational while the data were recorded.

Monte Carlo samples of simulated events are used to study the background modelling for the dominant $\gamma +\text {jet}$ processes, to optimize the selection criteria and to evaluate the acceptance and selection efficiencies for the signals considered in the search. Events from SM processes containing a photon with associated jets were simulated using the Sherpa  2.1.1 [24] event generator, requiring a photon transverse energy $E_{\text {T}} ^{\gamma }$ above 70 GeV at the generator level. The matrix elements were calculated with up to four final state partons at leading order ($\text {LO}$) in quantum chromodynamics (QCD) and merged with the parton shower  [25] using the ME+PS@LO prescription [26]. The CT10 [27] parton distribution function (PDF) set was used in conjunction with dedicated parton shower tuning developed by the Sherpa authors. A second sample of SM $\gamma +\text {jet}$ events was generated using the LO Pythia  8.186 [28] event generator with the LO NNPDF 2.3 PDFs [29] and the A14 tuning of the underlying-event parameters  [30]. The Pythia simulation includes leading-order $\gamma$ + jet events from both the direct processes (the hard subprocesses $qg \rightarrow q\gamma$ and $q\bar{q} \rightarrow g\gamma$) and bremsstrahlung photons in QCD dijet events. To estimate the systematic uncertainty associated with the background modelling, a large sample of events was generated with the next-to-leading-order (NLO) Jetphox  v1.3.1_2 [31] program. Events were generated at parton level for both the direct and fragmentation photon contributions using the NLO photon fragmentation functions [32] and the NLO NNPDF 2.3 PDFs, and setting the renormalization, factorization and fragmentation scales to the photon $E_{\text {T}} ^{\gamma }$. Jets of partons are reconstructed using the anti-$k_{t}$ algorithm [33, 34] with a radius parameter of $R=0.4$. The generated photon is required to be isolated by ensuring that the total transverse energy of partons inside a cone of size $\Delta R =0.4$ around the photon is smaller than 7.07 GeV + $0.03\times E_{\text {T}} ^{\gamma }$, equivalent^2^ to the photon selection for the data described in Sect. 4.

Samples of excited quark events were produced using Pythia  8.186 with the LO NNPDF 2.3 PDFs and the A14 set of tuned parameters for the underlying event. The Standard Model gauge interactions and the magnetic-transition type couplings [1–3] to gauge bosons were considered in the production processes of the excited states of the first-generation quarks ($u^*$, $d^*$) with degenerate masses. The compositeness scale $\Lambda$ was taken to be equal to the mass $m_{q^*}$ of the excited quark, and the coefficients $f_\mathrm {s}$, *f* and $f'$ of magnetic-transition type couplings to the respective SU(3), SU(2) and U(1) gauge bosons were chosen to be unity. The $q^*$ samples were generated with $m_{q^*}$ values between 0.5 and 6.0 TeV in steps of 0.5 TeV.

The QBH samples were generated using the QBH 2.02 [35] event generator with the CTEQ6L1 [36] PDF set and Pythia 8.186 for the parton shower and underlying event tuned with the A14 parameter set. The $M_{\text {th}}$ values were chosen to vary between 3.0 (1.0) and 9.0 (7.0) TeV in steps of 0.5 TeV for the QBH signals in the ADD (RS1) model. All the *qg*, $\bar{q}g$, *gg* and $q\bar{q}$ processes were included in the QBH signal production while only final states with a photon and a quark/gluon were considered for the decay. All six quark flavours were included together with their anti-quark counterparts in both the production and decay processes.

Apart from the sample generated with Jetphox which is a parton-level calculator, all the simulated samples include the effects of multiple $pp$ interactions in the same and neighbouring bunch crossings (pile-up) and were processed through the ATLAS detector simulation [37] based on Geant4  [38]. Pile-up effects were emulated by overlaying simulated minimum-bias events from Pythia  8.186, generated with the A2 tune [39] for the underlying event and the MSTW2008LO PDF set  [40]. The number of overlaid minimum-bias events was adjusted to match the one observed in data. All the MC samples except for the Jetphox sample were reconstructed with the same software as that used for collision data.

## Event selection

Photons are reconstructed from clusters of energy deposits in the EM calorimeter as described in Ref. [41]. A photon candidate is classified depending on whether the EM cluster is associated with a conversion track candidate reconstructed in the ID. If no ID track is matched, the candidate is considered as an unconverted photon. If the EM cluster is matched to either a conversion vertex formed from two tracks constrained to originate from a massless particle or a single track with its first hit after the innermost layer of the pixel detector, the candidate is considered to be a converted photon. Both the converted and unconverted photon candidates are used in the analysis. The energy of each photon candidate is corrected using MC simulation and data as described in Ref. [42]. The EM energy clusters are calibrated separately for converted and unconverted photons, based on their properties including the longitudinal shower development. The energy scale and resolution of the photon candidates after the MC-based calibration are further adjusted based on a correction derived using $Z \rightarrow e^{+} e^{-}$ events from data and MC simulation. Photon candidates are required to have $E_{\text {T}} ^{\gamma } >25$ GeV and $|\eta ^{\gamma } |<2.37$ and satisfy the “tight” identification criteria defined in Ref. [41]. Photons are identified based on the profile of the energy deposits in the first two layers of the EM calorimeter and the energy leakage into the hadronic calorimeter. To further reduce the contamination from $\pi ^{0}$ $\rightarrow \gamma \gamma$ or other neutral hadrons decaying into photons, the photon candidates are required to be isolated from other energy deposits in an event. The calorimeter isolation variable $E_{\text {T,\,iso}}$ is defined as the sum of the $E_{\mathrm {T}}$ of all positive-energy topological clusters [43] reconstructed within a cone of $\Delta R = 0.4$ around the photon direction excluding the energy deposits in an area of size $\Delta \eta \times \Delta \phi =0.125 \times 0.175$ centred on the photon cluster. The photon energy expected outside the excluded area is subtracted from the isolation energy while the contributions from pile-up and the underlying event are subtracted event by event [44]. The photon candidates are required to have $E_{\text {T,\,iso}}^{\gamma } =E_{\text {T,\,iso}}-0.022\times E_{\text {T}} ^{\gamma }$ less than 2.45 GeV. This $E_{\text {T}} ^{\gamma }$-dependent selection requirement is used to guarantee an efficiency greater than 90% for signal photons in the $E_{\text {T}} ^{\gamma }$ range relevant for this analysis. The efficiency for the signal photon selection varies from ($90\pm 1$)% to ($83\pm 1$)% for signal events with masses from 1 to 6 TeV. The dependency on the signal mass is mainly from the efficiency of the tight identification requirement while the isolation selection efficiency is approximately ($99\pm 1$)% over the full mass range.

Jets are reconstructed from topological clusters calibrated at the electromagnetic scale using the anti-$k_{t}$ algorithm with a radius parameter $R=0.4$. The jets are calibrated to the hadronic energy scale by applying corrections derived from MC simulation and in situ measurements of relative jet response obtained from *Z*+jets, $\gamma$+jets and multijet events at $\sqrt{s}$ = 13 TeV [45–47]. Jets from pile-up interactions are suppressed by applying the jet vertex tagger [48], using information about tracks associated with the hard-scatter and pile-up vertices, to jets with $p_{\text {T}} ^\text {jet} <60$ GeV and $|\eta ^{\text {jet}} |<2.4$. In order to remove jets due to calorimeter noise or non-collision backgrounds, events containing at least one jet failing to satisfy the loose quality criteria defined in Ref. [49] are discarded. Jets passing all the requirements and with $p_{\text {T}} ^\text {jet} >20$ GeV and $|\eta ^{\text {jet}} |<4.5$ are considered in the rest of the analysis. Since a photon is also reconstructed as a jet, jet candidates in a cone of $\Delta R = 0.4$ around a photon are not considered.

This analysis selects events based on a single-photon trigger requiring at least one photon candidate with $E_{\text {T}} ^{\gamma }>140$ GeV which satisfies loose identification conditions [41] based on the shower shape in the second sampling layer of the EM calorimeter and the energy leakage into the hadronic calorimeter. Selected events are required to contain at least one primary vertex with two or more tracks with $p_{\text {T}} >400$ MeV. Photon candidates are required to satisfy the “tight” identification and isolation conditions discussed above. The kinematic requirements for the highest-$E_{\text {T}}$ photon in the events are tightened to $E_{\text {T}} ^{\gamma } >150$ GeV and $|\eta ^{\gamma } |<1.37$. The $E_{\text {T}} ^{\gamma }$ requirement is used to select events with nearly full trigger efficiency [50] while the $\eta ^{\gamma }$ requirement is imposed to enhance the signal-to-background ratio. Moreover, an event is rejected if there is any jet with $p_{\text {T}} ^\text {jet} >30$ GeV within $\Delta R <0.8$ around the photon. The presence of additional tight and isolated photons with $E_{\text {T}} ^{\gamma }>150$ GeV in events is negligible for both signal and background events, and therefore allowed. The $\gamma +\text {jet}$ system is formed from the highest-$E_{\text {T}}$ photon and the highest-$p_{\text {T}}$ jet in the event. Finally, the highest-$p_{\text {T}}$ jet in the event is required to have $p_{\text {T}} ^\text {jet} >60$ GeV and the pseudorapidity difference between the photon and the jet ($\Delta \eta _{\gamma j} \equiv |\eta ^{\gamma }-\eta ^{\text {jet}} |$) must be less than 1.6 to enhance signals over the $\gamma +\text {jet}$ background, which typically has a large $\Delta \eta _{\gamma j}$ value. After applying all the selection requirements, $6.34\times 10^{5}$ events with an invariant mass ($m_{\gamma j}$) of the selected $\gamma +\text {jet}$ system greater than 500 GeV remain in the data sample.

## Statistical analysis

The data are examined for the presence of a significant deviation from the SM prediction using a test statistic based on a profile likelihood ratio  [51]. Limits on the visible cross-section for generic Gaussian-shaped signals and limits on the cross-section times branching ratio for specific benchmark models are computed using the CL$_{\text {S}}$ prescription  [52]. The details of the signal and background modelling used for the likelihood function construction are discussed in Sects. 5.1 and 5.2 while a summary of the statistical procedures used to establish the presence of a signal or set limits on the production cross-sections for new phenomena is given in Sect. 5.3.

### Signal modelling

The signal model is built starting from the probability density function (pdf), $f_{\text {sig}}(m_{\gamma j})$, of the $m_{\gamma j}$ distribution at the reconstruction level. For a Gaussian-shaped resonance with mass $m_{\text {G}}$, the $m_{\gamma j}$ pdf is modelled by a normalized Gaussian distribution with the mean located at $m_{\gamma j} = m_{\text {G}}$. The standard deviation of the Gaussian distribution is chosen to be 2, 7 or 15% of $m_{\text {G}}$, where 2% approximately corresponds to the effect of the detector resolution on the reconstruction of the photon–jet invariant mass. For the $q^*$ and QBH signals, the $m_{\gamma j}$ pdfs are created from the normalized reconstructed $m_{\gamma j}$ distributions after applying the selection requirements described in Sect. 4 using the simulated MC events, and a kernel density estimation technique [53] is applied to smooth the distributions. The signal pdfs for intermediate mass points at which signal events were not generated are obtained from the simulated signal samples by using a moment-morphing method [54]. The signal template for the $q^*$ and QBH signals is then constructed as $f_{\text {sig}}(m_{\gamma j}) \times (\sigma \cdot B \cdot A \cdot \varepsilon ) \times \mathcal {L}_{\text {int}}$, where the $f_{\text {sig}}$ is scaled by the product of the cross-section times branching ratio to a photon and a quark or gluon ($\sigma \cdot {B}$), acceptance (*A*), selection efficiency ($\varepsilon$) and the integrated luminosity ($\mathcal {L}_{\text {int}}$) for the data sample. The product of the acceptance times efficiency ($A \cdot \varepsilon$) is found to be about 50% for all the $q^*$ and QBH models, varying only by a few percent with $m_{q^*}$ or $M_{\text {th}}$. This dependence is accounted for in the model by interpolating between the generated mass points using a third-order spline. For the $q^*$ and QBH signals, limits are set on $\sigma \cdot {B}$ after correcting for the acceptance and efficiency $A \cdot \varepsilon$ of the selection criteria.

Experimental uncertainties in the signal yield arise from uncertainties in the luminosity ($\pm 3.2\%$), photon identification efficiency ($\pm 2\%$), trigger efficiency ($\pm 1\%$ as measured in Ref. [50] ) and pile-up dependence ($\pm 1\%$). The impact of the uncertainties in the photon isolation efficiency, photon and jet energy scales and resolutions is negligible. A 1% uncertainty in the signal yield is included to account for the statistical error in the acceptance and selection efficiency estimates due to the limited size of the MC signal samples. The impact of the PDF uncertainties on the signal acceptance is found to be negligible compared to the other uncertainties. The photon and jet energy resolution uncertainties ($\pm 2\%$ of the mass) are accounted for as a variation of the width for the Gaussian-shaped signals. The impact of the resolution uncertainty on intrinsically large width signals is found to be negligible and thus not included in the signal models for the $q^*$ and QBH. The typical difference between the peaks of the reconstructed and generator-level $m_{\gamma j}$ distributions for the excited-quark signals is well below 1%.

A summary of systematic uncertainties in the signal yield and shape included in the statistical analysis is given in Table 1.

**Table 1:** Summary of systematic uncertainties in the signal event yield and shape included in the fit model. The signal mass resolution uncertainty affects the generic Gaussian signal shape, while the other uncertainties affect the event yield

Uncertainty	$q^*$ and QBH	Generic Gaussian
Signal mass resolution	N/A	$\pm 2\% \cdot m_{\mathrm {G}}$
Photon identification	$\pm 2\%$	N/A
Trigger efficiency	$\pm 1\%$	N/A
Pile-up dependence	$\pm 1\%$	N/A
MC event statistics	$\pm 1\%$	N/A
Luminosity	$\pm 3.2\%$	

In order to facilitate the re-interpretation of the present results in alternative physics models, the fiducial acceptance and efficiency for events with the invariant mass of the $\gamma$ + jet system around $m_{q^*}$ or $M_{\text {th}}$ (referred to as “on-shell” events hereafter) are also provided. The chosen $m_{\gamma j}$ ranges are $0.6m_{q^*}<m_{\gamma j} <1.2m_{q^*}$ for the $q^*$ signal and $0.8M_{\text {th}}<m_{\gamma j} <3.0M_{\text {th}}$ for the QBH signal. The fiducial region at particle level, as summarized in Table 2, is chosen to be close to the one used in the event selection at reconstruction level.

**Table 2:** Requirements on the photon and jet at particle level to define the fiducial region and on the detector-level quantities for the selection efficiency

Particle-level selection for fiducial region	
Photon: $E_{\text {T}} ^{\gamma } >150$ GeV, $|\eta ^{\gamma } |<1.37$	
Jet: $p_{\text {T}} ^\text {jet} >60$ GeV, $|\eta ^{\text {jet}} |<4.5$	
Photon–Jet $\eta$ separation: $|\Delta \eta _{\gamma j} |<1.6$	
No jet with $p_{\text {T}} ^\text {jet} >30$ GeV within $\Delta R <0.8$ around the photon	
Detector-level selection for selection efficiency	
Tight photon identification	
Photon isolation	
Jet identification including quality and pile-up rejection requirements	

The fiducial acceptance $A_\mathrm {f}$, defined as the fraction of generated on-shell signal events falling into the fiducial region, increases from 56 to 63% with increasing signal mass $M_{\text {th}}$ from 1.0 to 6.5 (9.0) TeV for the QBH in the RS1 (ADD) model. The $A_\mathrm {f}$ value for the $q^*$ model varies very similarly to that for the RS1 QBH signal. The rise in the fiducial acceptance as a function of $M_{\text {th}}$ ($m_{q^*}$) is driven mainly by the increase of the efficiency for the photon $\eta$ requirement since the photons tend to be more central as $M_{\text {th}}$ ($m_{q^*}$) becomes larger.

**Fig. 1:**
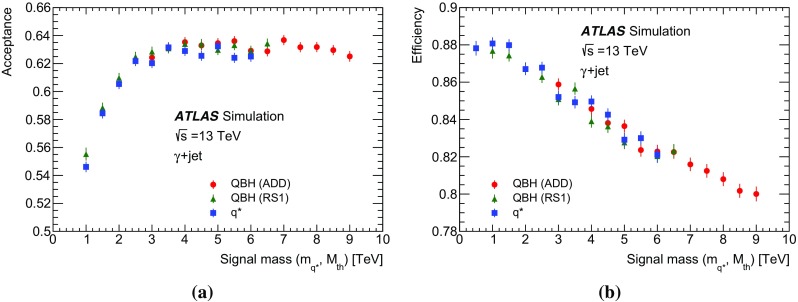
**a** Fiducial acceptance and **b** selection efficiencies for the three signal models considered in the analysis as a function of the excited-quark mass $m_{q^*}$ or the QBH threshold mass $M_{\text {th}}$. The fiducial region definition is detailed in Table 2. The description of the selection criteria can be found in the text

The fiducial selection efficiency $\varepsilon _\mathrm {f}$ is defined as the ratio of the number of on-shell events in the particle-level fiducial region passing the selection at the reconstruction level, including photon identification, isolation and jet quality criteria, to the number of generated on-shell events in the particle-level fiducial region. The migration of generated on-shell events outside the particle-level fiducial region into the selected sample at the reconstruction level is found to be negligible. The fiducial selection efficiency decreases from 88 (86) to 82 (80)% within the same $M_{\text {th}}$ ranges as above for the RS1 (ADD) QBH model and is not highly dependent on the kinematics of the assumed signal production processes. The $\varepsilon _\mathrm {f}$ for the $q^*$ model behaves very similarly to that for the RS1 QBH model. The reduction in the fiducial selection efficiency is caused mainly by the inefficiency of the shower shape requirements used in the photon identification for high-$E_{\text {T}}$ photons. The fiducial acceptance and selection efficiencies for the three benchmark signal models are shown in Fig. 1 as functions of $m_{q^*}$ or $M_{\text {th}}$.

### Background modelling

The $m_{\gamma j}$ distribution of the background is modelled using a functional form. A family of functions, similar to the ones used in the previous searches for $\gamma +\text {jet}$  [13, 14, 16] and $\gamma \gamma$ resonances [55] as well as dijet resonances [17] is considered:

1 $$\begin{aligned} f_{\text {bg}}(x) = N (1 - x)^{p} x^{\sum _{i=0}^k a_i (\log x)^i}, \end{aligned}$$

where *x* is defined as $m_{\gamma j}/\sqrt{s}$, *p* and $a_{i}$ are free parameters, and *N* is a normalization factor. The number of free parameters describing the normalized mass distribution is thus $k+2$ with a fixed *N*, where *k* is the stopping point of the summation in Eq. (1). The normalization *N* as well as the shape parameters *p* and $a_{i}$ are simultaneously determined by the final fit of the signal plus background model to data. The goodness of a given functional form in describing the background is quantified based on the potential bias introduced in the fitted number of signal events. To quantify this bias the functional form under test is used to perform a signal + background fit to a large sample of background events built from the Jetphox prediction. The large Jetphox event sample is used for this purpose as the shape of the background prediction can be extracted with sufficiently small statistical uncertainty.

**Fig. 2:**
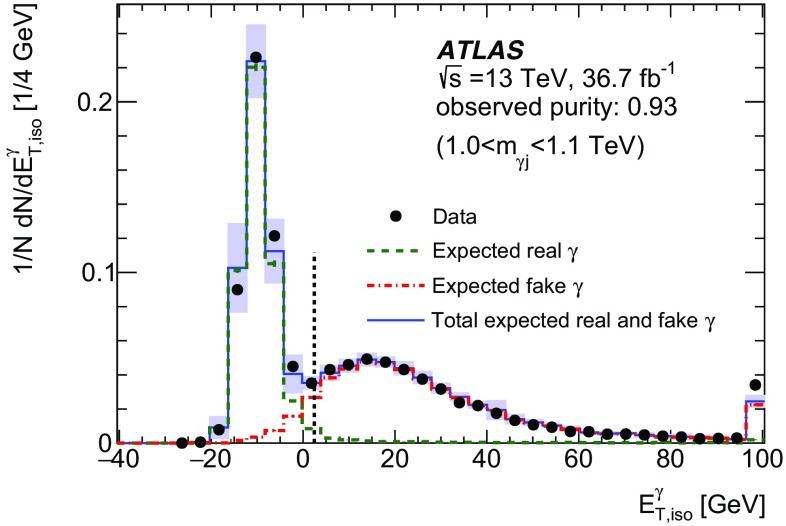
Distribution of $E_{\text {T,\,iso}}^{\gamma } =E_{\text {T,\,iso}}-0.022\times E_{\text {T}} ^{\gamma }$ for the photon candidates in events with $1.0<m_{\gamma j} <1.1$ TeV, and the comparison with the result of the template fit. Real- and fake-photon components determined by the fit are shown by the green dashed and red dot-dashed histograms, respectively, and the sum of the two components is shown as the solid blue histogram. The blue band shows the systematic uncertainties in the real- plus fake-photon template. The last bin of the distribution includes overflow events. The vertical dashed line corresponds to the isolation requirement used in the analysis. The photon purity determined from the fit for the selected sample in the $1.0<m_{\gamma j} <1.1$ TeV mass range is 93%

**Fig. 3:**
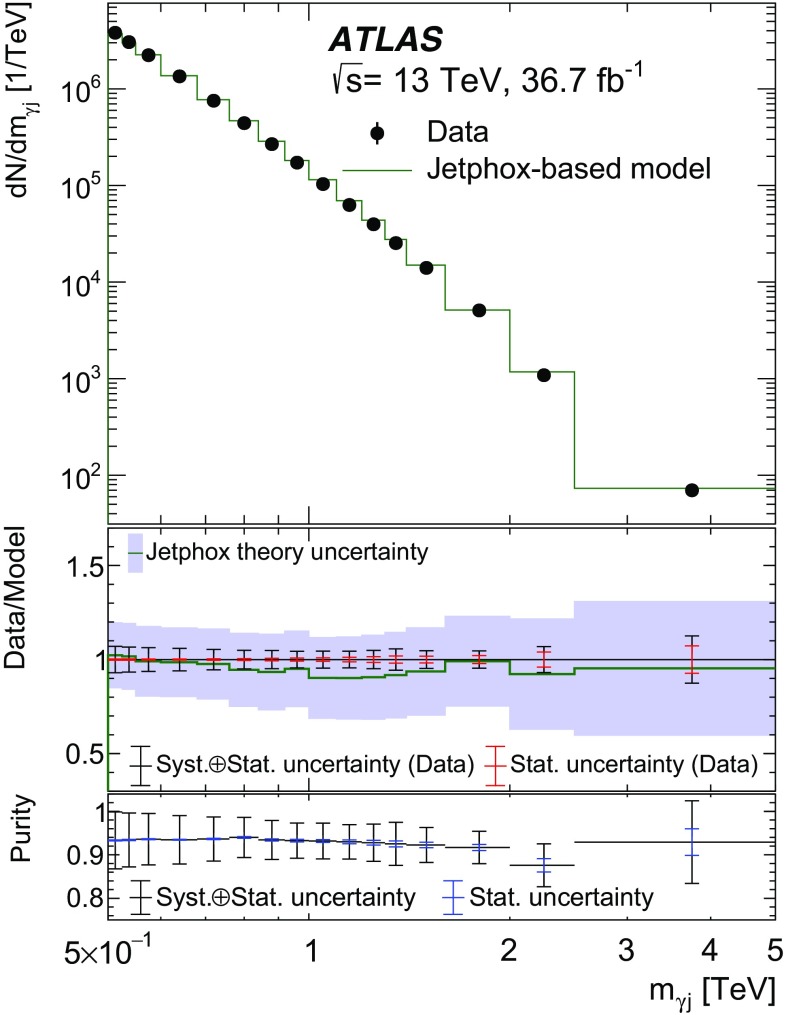
Distribution of the invariant mass of the $\gamma$ + jet system as measured in the $\gamma +\text {jet}$ data (dots), compared with the Jetphox (green histogram) $\gamma +\text {jet}$ predictions. The Jetphox distribution is obtained after correcting the parton-level spectrum for showering, hadronization and detector resolution effects as described in the text. The distributions are divided by the bin width and the Jetphox spectrum is normalized to the data in the $m_{\gamma j}$ range above 500 GeV. The ratio of the data to Jetphox prediction as a function of $m_{\gamma j}$ is shown in the middle panel (green histogram): the theoretical uncertainty is shown as a shaded band. The statistical uncertainty from the data sample and the sum of the statistical uncertainty plus the systematic uncertainty from the background subtraction are shown as inner and outer bars respectively. The measured $\gamma +\text {jet}$ purity as a function of $m_{\gamma j}$ is presented in the bottom panel (black histogram): the statistical uncertainty of the purity measurement is reported as the inner error bar while the total uncertainty is shown as the outer error bar

The parton-level Jetphox calculations do not account for effects from hadronization, the underlying event and the detector resolution. Therefore, the nominal Jetphox prediction is corrected by calculating the ratio of reconstructed jet $p_{\text {T}}$ to parton $p_{\text {T}}$ in the Sherpa $\gamma +\text {jet}$ sample and applying the parameterized ratio to the Jetphox parton $p_{\text {T}}$. In addition, an $m_{\gamma j}$-dependent correction is applied to the Jetphox prediction to account for the contribution from multijet events where one of the jets is misidentified as a photon (fake photon events). This correction is estimated from data as the inverse of the purity, defined as the fraction of real $\gamma +\text {jet}$ events in the selected sample. The purity is measured in bins of $m_{\gamma j}$ by exploiting the difference between the shapes of the $E_{\text {T,\,iso}}^{\gamma }$ distributions of real photons and jets faking photons; the latter typically have a large $E_{\text {T,\,iso}}^{\gamma }$ value due to nearby particles produced in the jet fragmentation. The purity is estimated by performing a two-component template fit to the $E_{\text {T,\,iso}}^{\gamma }$ distribution in bins of $m_{\gamma j}$. The templates of real- and fake-photon isolation distributions are obtained from MC (Sherpa) simulation and from data control samples, respectively. The $E_{\text {T,\,iso}}^{\gamma }$ variable for real photons from Sherpa simulation is corrected to account for the observed mis-modelling in the description of isolation profiles between data and MC events in a separate control sample. The template for fake photons is derived in a data sample where the photon candidate fails to satisfy the tight identification criteria but fulfils a looser set of identification criteria. Details about the correction to the real-photon template and the derivation of the fake-photon template are given in Ref. [56]. To reduce the bias in the $E_{\text {T,\,iso}}^{\gamma }$ shape due to different kinematics, both the real- and fake-photon templates are obtained by applying the same set of kinematic requirements used in the main analysis. As an example, Fig. 2 shows the $E_{\text {T,\,iso}}^{\gamma }$ distribution of events within the range $1.0<m_{\gamma j} <1.1$ TeV, superimposed on the best-fit result. This procedure is repeated in every bin of the $m_{\gamma j}$ distribution and the resulting estimate of the purity is shown as a function of $m_{\gamma j}$ in Fig. 3. The uncertainty in the measured purity includes both the statistical and systematic uncertainties. The latter are estimated by recomputing the purity using different data control samples for the fake-photon template or alternative templates for real photons obtained from Pythia simulation or removing the data-to-MC corrections applied to $E_{\text {T,\,iso}}^{\gamma }$ in the Sherpa sample and by symmetrizing the variations. The variation from different data control samples for the fake-photon template has the largest effect on the purity (4% at $1.0< m_{\gamma j} < 1.1$ TeV). The measured purity is approximately constant at 93% over the $m_{\gamma j}$ range above 500 GeV, indicating that the fake-photon contribution does not depend significantly on $m_{\gamma j}$. Figure 3 shows the $m_{\gamma j}$ distribution in data compared to the corrected Jetphox $\gamma +\text {jet}$ prediction normalized to data in the $m_{\gamma j} > 500$ GeV region.

Theoretical uncertainties in the Jetphox prediction are computed by considering the variations induced by $\pm 1\sigma$ of the NNPDF 2.3 PDF uncertainties, by switching between the nominal NNPDF 2.3 and CT10 or MSTW2008 PDFs, by the variation of the value of the strong coupling constant by $\pm 0.002$ around the nominal value of 0.118 and by the variation of the renormalization, factorization and fragmentation scales between half and twice the photon transverse momentum. The differences between data and the corrected Jetphox prediction shown in Fig. 3 are well within the uncertainties associated with the perturbative QCD prediction.

The number of signal events extracted by the signal + background fit to the pure background model described above is called the *spurious signal*  [57] and it is used to select the optimal functional form and the $m_{\gamma j}$ range of the fit. In order to account for the assumption that the corrected Jetphox prediction itself is a good representation of the data, the fit is repeated on modified samples obtained by changing the nominal shape to account for several effects: firstly, the nominal distribution is corrected to follow the envelope of the changes induced by $\pm 1\sigma$ variations of the NNPDF 2.3 PDF uncertainty, the variations between the nominal NNPDF 2.3 and CT10 or MSTW2008 PDFs, the variation of the value of the strong coupling constant by $\pm 0.002$ around the nominal value of 0.118 and the variation of the renormalization, factorization and fragmentation scales between half and twice the photon transverse momentum; secondly the corrections for the hadronization, underlying event and detector effects are removed; and finally the corrections for the photon purity are changed within their estimated uncertainty. The largest absolute fitted signal from all variations of the nominal background sample discussed above is taken to be the spurious signal.

The spurious signal is evaluated at a number of hypothetical masses over a large search range. It is required to be less than 40% of the background’s statistical uncertainty, as quantified by the statistical uncertainty of the fitted spurious signal, anywhere in the investigated search range. In this way the impact of the systematic uncertainties due to background modelling on the analysis sensitivity is expected to be subdominant with respect to the statistical uncertainty. Functional forms that cannot meet this requirement are rejected. For different signal models, the functional form and fit range are determined separately. All considered functions with *k* up to two (four parameters) are found to fulfil the spurious-signal requirement when fitting in the range $1.1<m_{\gamma j} <6.0$ TeV for the $q^*$ signal and $1.5~(2.5)<m_{\gamma j} <6.0~(8.0)$ TeV for the RS1 (ADD) QBH signal. To further consolidate the choice of nominal background functional form, an *F* test [58] is performed to determine if the change in the $\chi ^2$ value obtained by fitting the Jetphox sample with an additional parameter is significant. The test indicates that the $k=0$ (1) functional form with two (three) parameters can describe the present data sufficiently well over the entire fit range for the QBH ($q^*$) signal search, and there is no improvement by adding more parameters to the background fit function.

Given the fit range determined by the spurious signal test, the search is performed for the $q^*$ (RS1 and ADD QBH) signal within the $m_{\gamma j}$ range above 1.5 (2.0 and 3.0) TeV, to account for the width of the expected signal. The estimated spurious signal for the selected functional form is converted into a spurious-signal cross-section ($\sigma _{\text {spur}}$), which is included as the uncertainty due to background modelling in the statistical analysis. The spurious-signal cross-section, and the ratio of the spurious-signal cross-section to its uncertainty ($\delta \sigma _{\mathrm {spur}}$) and to the signal cross-section ($\sigma _{\text {model}}$) for the three benchmark models under investigation are given in Table 3 in the different search ranges. While both $\sigma _{\text {spur}}$ and $\sigma _{\text {spur}}$/$\delta \sigma _{\mathrm {spur}}$ decrease with the hypothesized signal mass, the ratio $\sigma _{\text {spur}}$/$\sigma _{\text {model}}$ increases with $m_{q^*}$ or $M_{\text {th}}$, becoming as large as 15% in the case of excited quarks with $m_{q^*}$ = 6 TeV.

**Table 3:** Spurious-signal cross-sections ($\sigma _{\text {spur}}$), and the ratio of the spurious-signal cross-sections to their uncertainties ($\delta \sigma _\mathrm {spur}$) and to the signal cross-sections ($\sigma _{\text {model}}$) for the three benchmark models. The values of these quantities are given at the boundaries of the search range reported in the first row

	$q^*$		RS1 QBH		ADD QBH	
Search boundaries (TeV)	1.5	6.0	2.0	6.0	3.0	8.0
$\sigma _{\text {spur}}$ (fb)	3.9	$1.1\times 10^{-2}$	4.0	$6.6\times 10^{-4}$	$8.7\times 10^{-2}$	$5.0\times 10^{-5}$
$\sigma _{\text {spur}}$/ $\delta \sigma _\mathrm {spur}$ (%)	37	14	39	8	20	3
$\sigma _{\text {spur}}$/$\sigma _{\text {model}}$ (%)	0.16	15	1.0	7.5	0.0017	0.037

A similar test is performed to determine the functional form and fit ranges for the Gaussian-shaped signal with a 15% width. The test indicates that the same functional form and fit range as those used for the $q^*$ signal are optimal for a wide-width Gaussian signal. The same functional form and mass range is used for all the Gaussian signals.

### Statistical tests

A profile-likelihood-ratio test statistic is used to quantify the compatibility between the data and the SM prediction, and to set limits on the presence of possible signal contributions in the $m_{\gamma j}$ distribution. The likelihood function $\mathcal {L}$ is built from a Poisson probability for the numbers of observed events, *n*, and expected events, *N*, in the selected sample:

$$\begin{aligned} \mathcal {L} = \text {Pois}(n|N(\varvec{\theta })) \times \left( \prod _{i=1}^n f(m_{\gamma j} ^i,\varvec{\theta })\right) \times G(\varvec{\theta }), \end{aligned}$$

where $N(\varvec{\theta })$ is the expected number of candidates, $f(m_{\gamma j} ^i, \varvec{\theta })$ is the value of the probability density function of the invariant mass distribution evaluated for each candidate event *i* and $\varvec{\theta }$ are nuisance parameters. The $G(\varvec{\theta })$ term collects the set of constraints on the nuisance parameters associated with the systematic uncertainties in the signal yield, in the spurious signal and in the resolution (only for Gaussian signals) and it is represented by normal distributions centred at zero and with unit variance.

The pdf of the $m_{\gamma j}$ distribution is given as the normalized sum of the signal and background pdfs:

$$\begin{aligned} f(m_{\gamma j} ^i,\varvec{\theta }) = \frac{1}{N} \left[ N_{\text {sig}} (\varvec{\theta } _{\mathrm {yield}}) f_{\text {sig}}(m_{\gamma j} ^i) + N_{\text {bg}} f_{\text {bg}}(m_{\gamma j} ^i,\varvec{\theta } _{\mathrm {bkg}})\right] , \end{aligned}$$

where $f_{\text {sig}}$ and $f_{\text {bg}}$ are the normalized signal and background $m_{\gamma j}$ distributions described in the previous sections. The $\varvec{\theta } _{\mathrm {yield}}$ are nuisance parameters associated with the signal yield uncertainties (constrained) while $\varvec{\theta } _{\mathrm {bkg}}$ are the nuisance parameters of the background shape (unconstrained). The expected number of events *N* is given by the sum of the expected numbers of signal events ($N_{\text {sig}}$) and background events ($N_{\text {bg}}$). The $N_{\text {sig}}$ term can be expressed as

$$\begin{aligned} N_{\text {sig}} (\varvec{\theta } _{\mathrm {yield}})= & {} N_{\text {sig}}^{\text {model}} + N_{\text {sig}}^{\text {spur}} \\= & {} (\sigma _{\text {model}} \cdot B \cdot A \cdot \varepsilon \cdot F(\varvec{\delta }_{\varepsilon },\varvec{\theta }_{\varepsilon })+ \sigma _{\text {spur}} \cdot \theta _{\text {spur}}) \nonumber \\&\times \mathcal {L}_{\text {int}} \times F(\delta _{\mathcal {L}},\theta _{\mathcal {L}}), \end{aligned}$$

where $\sigma _{\text {spur}}$ and $\theta _{\text {spur}}$ are the spurious-signal cross-section described in Sect. 5.2 and its nuisance parameter while $\mathcal {L}_{\text {int}}$ and $F(\delta _{\mathcal {L}},\theta _{\mathcal {L}})$ are the integrated luminosity and its uncertainty. Apart from the spurious signal, systematic uncertainties with an estimated size $\delta _{X}$ are incorporated into the likelihood by multiplying the relevant parameter of the statistical model by a factor $F(\delta _{X},\theta _{X})=\mathrm {e}^{\delta _{X} \theta _{X}}$. The parameter of interest in the fit to Gaussian-shaped resonances is the visible cross-section $\sigma _{\text {model}} \cdot B \cdot A \cdot \varepsilon$ while that in the fit to $q^*$ and QBH signals is $\sigma _{\text {model}} \cdot B$. For the latter case, the additional nuisance parameters for the signal efficiency uncertainties $F(\varvec{\delta }_{\varepsilon },\varvec{\theta }_{\varepsilon })$ are included.

The significance of a possible deviation from the SM prediction is estimated by computing the $p_0$ value, defined as the probability to observe, under the background model hypothesis, an excess at least as large as the one observed in data. Upper limits are set at 95% confidence level (CL) with a modified frequentist CL$_{\text {S}}$ method on the visible cross-section ($\sigma _{\text {model}} \cdot B \cdot A \cdot \varepsilon$) for the Gaussian-shaped resonances or on the signal cross-section times branching ratio ($\sigma _{\text {model}} \cdot B$) for the $q^*$ and QBH signals by identifying the value for which the CL$_{\text {S}}$ value is equal to 0.05.

## Results

**Fig. 4:**
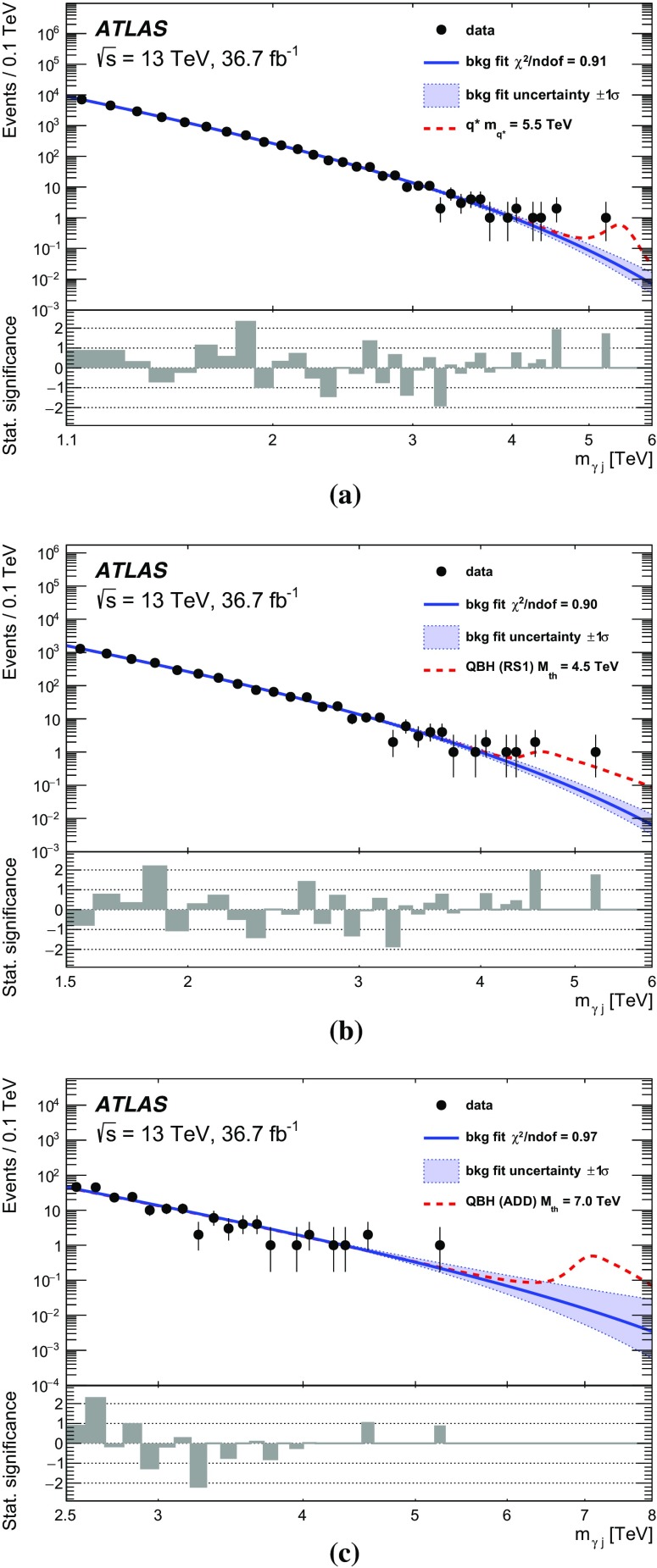
Distributions of the invariant mass of the $\gamma$ + jet system of the observed events (dots) in $36.7~\text {fb}^{-1}$ of data at $\sqrt{s}$ = 13 TeV and fits to the data (solid lines) under the background-only hypothesis for searches in the **a** excited quarks, **b** QBH (RS1) with $n=1$ and **c** QBH (ADD) with $n=6$ models. The $\pm 1\sigma$ uncertainty in the background prediction originating from the uncertainties in the fit function parameter values is shown as a shaded band around the fit. The predicted signal distributions (dashed lines) for the $q^*$ model with $m_{q^*}$ = 5.5 TeV and the QBH model with $M_{\text {th}}$ = 4.5 (7.0) TeV based on RS1 (ADD) are shown on top of the background predictions. The bottom panels show the bin-by-bin significances of the data–fit differences, considering only statistical uncertainties

The photon–jet invariant mass distributions obtained from the selected data are shown in Fig. 4, together with the background-only fits using the model described in Sect. 5.2 and expected distributions from the signal models under test. No significant deviation from the background prediction is observed in any of the distributions. The most significant excess is observed at 1.8 TeV with the assumption of the 2%-width Gaussian model for a local significance of 2.1 standard deviations.

**Fig. 5:**
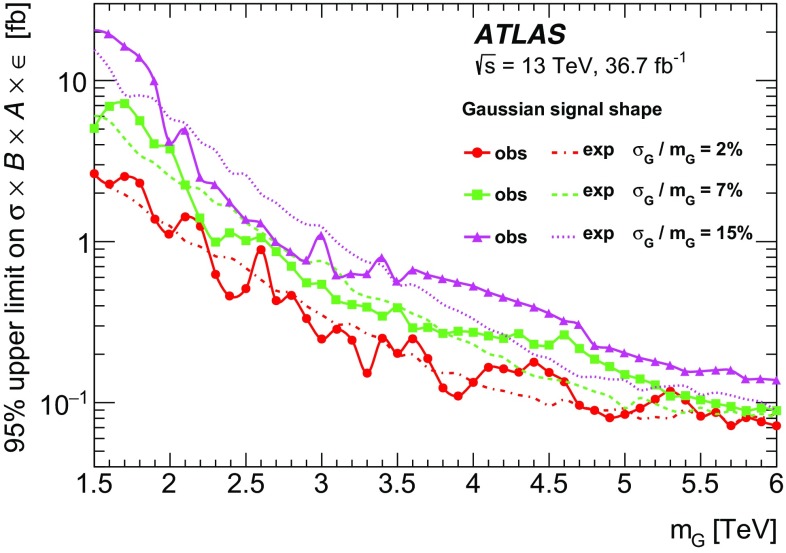
Observed (solid lines) and expected (dotted lines) 95% CL upper limits on the visible cross-sections $\sigma \cdot B \cdot A \cdot \varepsilon$ in $36.7~\text {fb}^{-1}$ of data at $\sqrt{s}$ = 13 TeV as a function of the mass $m_{\text {G}}$ of the Gaussian resonances with three different Gaussian widths between 2 and 15%. The calculation is performed using ensemble tests at mass points separated by 100 GeV over the search range

**Fig. 6:**
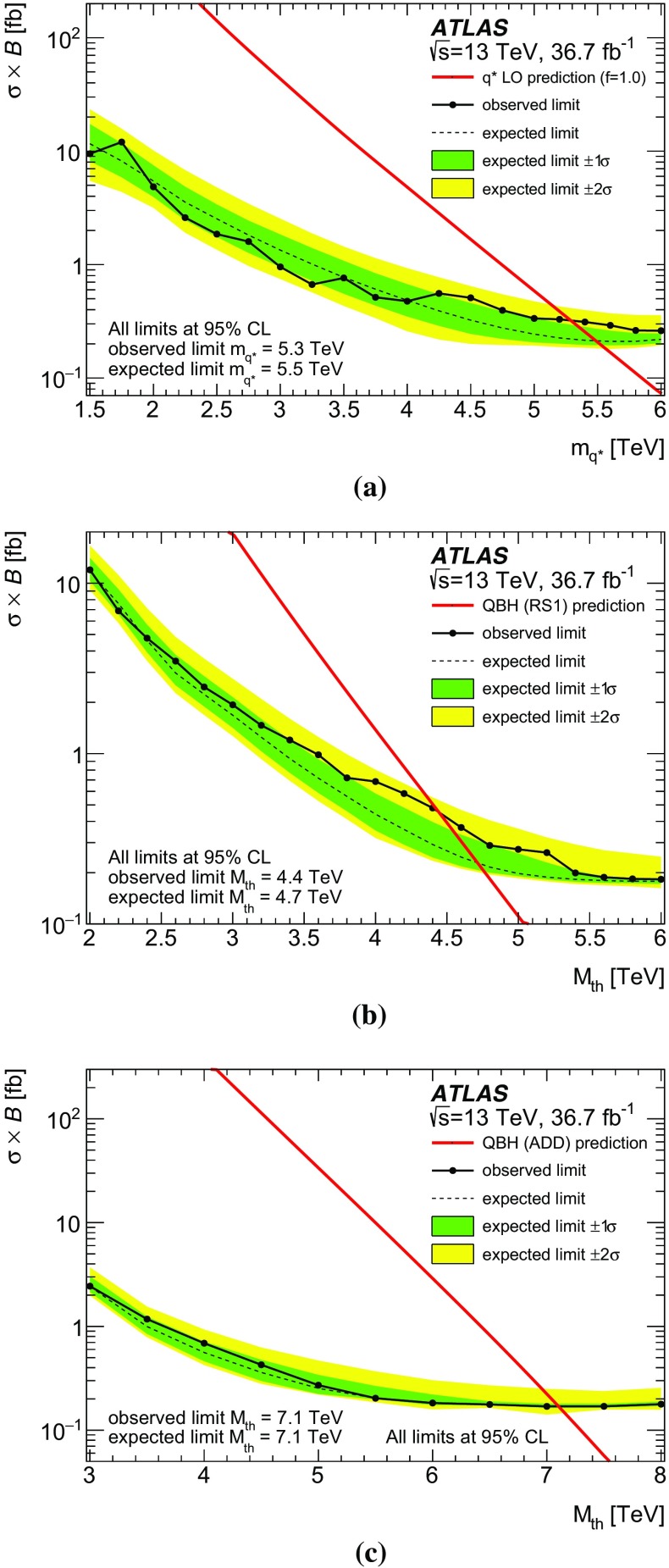
Observed 95% CL upper limits (solid line with dots) on the production cross-section times branching ratio $\sigma \cdot {B}$ to a photon and a quark or gluon in $36.7~\text {fb}^{-1}$ of data at $\sqrt{s}$ = 13 TeV for the **a** excited-quarks, **b** QBH (RS1) with $n=1$ and **c** QBH (ADD) with $n=6$ models. The limits are placed as a function of $m_{q^*}$ for the excited quarks and $M_{\text {th}}$ for the QBH signals. The calculation is performed using ensemble tests at mass points separated by 200 (500) GeV for the RS1 (ADD) model over the search range. For the $q^*$ model the step size is 250 GeV up to 5 TeV and then 200 GeV up to 6 TeV. The limits expected if a signal is absent (dashed lines) are shown together with the $\pm 1\sigma$ and $\pm 2\sigma$ intervals represented by the green and yellow bands, respectively. The theoretical predictions of $\sigma \cdot {B}$ for the respective benchmark signals are shown by the red solid lines.

Limits are placed at 95% CL on the visible cross-section in the case of generic Gaussian-shaped resonances and on the production cross-section times branching ratio to a photon and a quark or gluon for the excited-quark and QBH signals. The results are shown in Fig. 5 for the Gaussian signals with the width varying between 2 and 15%, and in Fig. 6 for the benchmark signal models. The Gaussian signals are excluded for visible cross-sections above 0.25–1.1 fb (0.08–0.2 fb), depending on the width, at a mass $m_{\text {G}}$ of 3 TeV (5 TeV). In the case of the benchmark signal models considered in this analysis, the presence of a signal with a mass below 5.3, 4.4 and 7.1 TeV for the excited quarks, RS1 and ADD QBHs, can be excluded at 95% CL. The limits improve on those in Ref. [16] by about 0.9, 0.6 and 0.9 TeV for the excited quarks, RS1 and ADD QBHs, respectively.

## Conclusion

A search is performed for new phenomena in events having a photon with high transverse momentum and a jet collected in $36.7~\text {fb}^{-1}$ of $pp$ collision data at a centre-of-mass energy of $\sqrt{s}$ = 13 TeV recorded with the ATLAS detector at the LHC. The invariant mass distribution of the $\gamma$ + jet system above 1.1 TeV is used in the search for localized excesses of events. No significant deviation is found. Limits are set on the visible cross-section for generic Gaussian-shaped resonances and on the production cross-section times branching ratio for signals predicted in models of excited quarks or quantum black holes. The data exclude, at 95% CL, the mass range below 5.3 TeV for the excited quarks and 7.1 (4.4) TeV for the quantum black holes with six (one) extra dimensions in the Arkani-Hamed–Dimopoulos–Dvali (Randall–Sundrum) model. These limits supersede the previous ATLAS exclusion limits for excited quarks and quantum black holes in the $\gamma +\text {jet}$ final state.

## Footnotes

1 ATLAS uses a right-handed coordinate system with its origin at the nominal interaction point (IP) in the centre of the detector and the *z*-axis along the beam pipe. The *x*-axis points from the IP to the centre of the LHC ring, and the *y*-axis points upwards. Cylindrical coordinates $(r,\phi )$ are used in the transverse plane, $\phi$ being the azimuthal angle around the *z*-axis. The pseudorapidity is defined in terms of the polar angle $\theta$ as $\eta = -\ln \tan (\theta /2)$. Angular distance is measured in units of $\Delta R \equiv \sqrt{(\Delta \eta )^{2} + (\Delta \phi )^{2}}$.

2 The parton-level isolation requirement takes into account the correlation between reconstruction-level isolation energies and particle-level isolation energies, as a proxy for the parton-level isolation, as evaluated using $\gamma +\text {jet}$ events simulated by Pythia  8.186.
